# The architecture of intra-organism mutation rate variation in plants

**DOI:** 10.1371/journal.pbio.3000191

**Published:** 2019-04-09

**Authors:** Long Wang, Yilun Ji, Yingwen Hu, Huaying Hu, Xianqin Jia, Mengmeng Jiang, Xiaohui Zhang, Lina Zhao, Yanchun Zhang, Yanxiao Jia, Chao Qin, Luyao Yu, Ju Huang, Sihai Yang, Laurence D. Hurst, Dacheng Tian

**Affiliations:** 1 State Key Laboratory of Pharmaceutical Biotechnology, School of Life Sciences, Nanjing University, Nanjing, China; 2 The Milner Centre for Evolution, Department of Biology and Biochemistry, University of Bath, Bath, United Kingdom; Institute of Science and Technology Austria (IST Austria), AUSTRIA

## Abstract

Given the disposability of somatic tissue, selection can favor a higher mutation rate in the early segregating soma than in germline, as seen in some animals. Although in plants intra-organismic mutation rate heterogeneity is poorly resolved, the same selectionist logic can predict a lower rate in shoot than in root and in longer-lived terminal tissues (e.g., leaves) than in ontogenetically similar short-lived ones (e.g., petals), and that mutation rate heterogeneity should be deterministic with no significant differences between biological replicates. To address these expectations, we sequenced 754 genomes from various tissues of eight plant species. Consistent with a selectionist model, the rate of mutation accumulation per unit time in shoot apical meristem is lower than that in root apical tissues in perennials, in which a high proportion of mutations in shoots are themselves transmissible, but not in annuals, in which somatic mutations tend not to be transmissible. Similarly, the number of mutations accumulated in leaves is commonly lower than that within a petal of the same plant, and there is no more heterogeneity in accumulation rates between replicate branches than expected by chance. High mutation accumulation in runners of strawberry is, we argue, the exception that proves the rule, as mutation transmission patterns indicate that runner has a restricted germline. However, we also find that in vitro callus tissue has a higher mutation rate (per unit time) than the wild-grown comparator, suggesting nonadaptive mutational “fragility”. As mutational fragility does not obviously explain why the shoot—root difference varies with plant longevity, we conclude that some mutation rate variation between tissues is consistent with selectionist theory but that a mechanistic null of mutational fragility should be considered.

## Abstract 2

The rates of accumulation of mutations vary between different parts of a plant, with lower rates when mutations have greater potential longevity (such as in leaves) than in short-lived structures (such as petals).

## Author summary

Whereas there has been considerable attention paid to understanding differences in the mutation rate between different species, much less is known about variation in the mutation rate within individuals of multicellular species. In animals, evidence suggests that the segregated germline has a lower mutation rate than somatic cells, which accords with an adaptive model of intra-organism mutation rate variation. Here, we consider related questions in plants, which, although not having an early segregated germline, present numerous opportunities to test such an adaptive model. In particular, such a model would predict a lower rate in shoots than in roots and in relatively long-lived leaves compared with ontogenetically related but more ephemeral petals. In addition, we expect that mutation rate variation should be deterministic, such that there is no more heterogeneity in mutation rates between similarly aged branches than expected by chance. By sequencing several hundred genomes for numerous different species, we find all predictions to be supported, with the proviso that the root—shoot difference is witnessed in perennials but not in an annual. This last difference we show to be explicable, as annuals transmit relatively few premeiotic mutations. Although the adaptive model is then parsimonious, we caution that the mutation rate in plants can be easily affected by local conditions, as evidenced by large differences between lab-grown callus and field-grown plants.

## Introduction

In some animals, the germline is segregated early in development, thereby preventing many (i.e., somatic) mutations from being transmitted to progeny [1]. Given this, classical theory of senescence posits that organisms have no vested interest in keeping the somatic mutation rate under control after the age of reproduction [2]. The same logic predicts that, given the reduced temporal longevity of any mutation, the shorter lived the organism, the higher the somatic mutation rate could be [3]. A higher somatic rate in mouse than human [4], for example, is consistent with such expectations. As the future potential longevity of new germline mutations is longer than that of somatic ones, special protection for the germline from mutation can sometimes be expected. In humans, for example, there is an unusually low per-cell-division mutation rate in the male germline [5]. More generally, somatic rates are typically reported to be higher than germline rates [4,6–9].

The extent to which such a theoretical framework, based on the potential longevity of a mutation, enables understanding of the variation in mutation rates between tissues across phyla is poorly understood, not least because of a dearth of data in many major groups (somatic mutation rates, either per cell division or per unit time, are for example hard to measure [10]). Whether plants are potentially informative in this debate is at first sight doubtful, not least because whether they have any clearly distinct soma and germline is contentious [11,12]. Leaving this uncertainty to one side, plants provide some relatively clear predictions and exceptional opportunities. We might, for example, expect a difference between root and stem, as stem alone has the prospect of being a germline progenitor. The transmissibility of mutations is not the only issue, however. Whereas germline mutations have a high prospective temporal longevity (i.e., they can be passed to the next generation and thence onwards), prospective longevity also varies between nongermline tissues. For example, petals and leaves, although ontogenetically related, have different longevities, the petal being highly transient and thus potentially under reduced selection to minimize mutation rates. Similarly, perennials and annuals will differ in the longevity of true somatic mutations and might differ in the proportion of premeiotic mutations that are transmissible. If so, annuals and perennials may differ also in the extent to which the shoot might have a reduced mutation rate, much as short- and long-lived mammals differ in their somatic rates [4].

Any model of selectively optimized mutation rate differences also predicts that variation between samples is not simply owing to stochastic variation. Plants provide excellent opportunities to test this given their branched structure and hence numerous biological replicates whose age can be ascertained. In this context, although we expect different branches to harbor different mutations [13–16], we do not necessarily expect some branches to be significantly more or less mutagenic than others; i.e., we expect heterogeneity to be between different tissues, not between biological replicates of the same tissues. The development of multiple branches in any growing season from the same plant permits an unusually well-controlled resource to test for homogeneity of mutation rates.

Between-branch differences are also important because bud breeding has long been a classical way to establish a good variety in perennial crops, especially in the important fruits and ornamental plants [17–19]. Indeed, the plant has long been viewed as a metapopulation [20], in which each branch evolves independently such that interbranch variation could prevent pest populations from adapting to all branches on individual host trees [21]. In this context as well, understanding the nature of between-branch mutation accumulation heterogeneity is of importance.

Above, we presume one model for between-tissue differences in mutation rate, this being a model in which the variation is understood as the product of selection on the rate of mutation accumulation. An alternative possibility is that the mutation rate is “fragile”—i.e., easily perturbed by, for example, intra-organismic local environment or growth conditions [22], but not necessarily in a selectively advantageous manner. If so, we might expect that the mutation rate of artificial callus tissue, raised in the lab, might be mutationally different from a field-grown comparator.

Here then, we attempt to define an extensive platform for consideration of the architecture of intra-organismic mutation accumulation in plants. To this end, across numerous species we ask whether roots and shoots have the same mutation rates, whether leaves and petals have different rates, whether we can detect between-branch heterogeneity, and whether tissue culturing modifies mutation rates. Consistent with an adaptive model, we find that roots commonly have higher mutation rates (per unit time) than shoots in perennials but not in an annual, petals have higher rates than leaves, and variation is between tissues rather than between biological replicates. However, we also find that callus has very high mutation rates. In addition, we assume that the apparent differences in mutation rate are just that and are not owing to differential degrees of purifying selection. We test for this possibility but find no support for it. In sum, the in vivo evidence is largely consistent with an adaptive mutation rate model, but in vitro data support the viability of a mechanistic null of context-dependent mutational fragility.

Note that we make no attempt to directly measure the per-cell-division rate, as the issue at stake is the net mutation accumulation. That is, a system that reduces the number of cell divisions in, for example, germline but not the per-cell-division mutation rate can be of equivalent selective consequence as one that reduces the per-division rate but not the number of such divisions. In this context, we measure net mutation accumulation with the “rates” being comparable between comparators but not necessarily defined in absolute terms (i.e., not per cell division).

## Results

### A higher mutation rate in root compared with shoot is common in perennial species

Prima facie, a selectionist model of mutation rate adaptation might predict a lower rate of mutation in stem compared with root, as mutations (which are mostly deleterious) are more likely to be transmissible to a subsequent generation if they occur in shoot. One could indeed argue that the root—shoot difference is the closest many plants get to anything resembling an unambiguous soma—germline distinction. Moreover, despite a distinct phenotype, the shoot and root share much in common about their organization of stem cell niches [12,23], which also resembles that of animal stem cell niches [23].

New leaf and new root at the terminal branch in a perennial plant are the best organs for comparison because they have an equal separation age (i.e., time to common cell ancestor in the embryo). If all else is equal, the average mutation number per leaf sample (or part leaf with a total of >80 mg for genome sequencing) should be comparable with the number per root sample with a similar weight from the same plant. Note that this assumes that their cell sizes are similar, and hence, a similar number of cells are sampled. We note that 200–500 ng of DNA per sample is used in library preparation and is sequenced. This ensures that even in tissues where DNA is hard to extract (e.g., root) or where the ratio of DNA mass to tissue mass is low, the total amount of sequenced DNA is approximately invariant.

Four pairs of leaf and root samples were collected from three perennials (*Prunus persica*, *P*. *mume*, *Salix suchowensis*) and one annual (*Brachypodium distachyon*) species (Table 1). A total of 96 leaf and 74 root samples were sequenced. Mutations were called following stringent quality control and by reference to the ancestral state derived by the allelic state elsewhere in the same plant (S1 Fig). To minimize miscalling, we employ two different calling methods and require mutations to either be called by both or, if called by just one, to be confirmed by other means (see Materials and methods).

**Table 1:** Accumulated somatic mutations per sample in each terminal branch of all sequenced plant samples.

Species (reference genome size)	Samples	Diameter of the trunk (cm)	Estimated age (years)	DNA source	Sequenced samples	Accumulated mutations	
						Average observed	Normalized rate (× 10^−9^ per bp per year)^a^
*P*. *mira* (225 Mb)	G1^b^	207	600	Leaf	32	12.7	0.08
	G2	191	550	Leaf	12	23.9	0.15
	GL2	148	420	Leaf	23	17.7	0.14
	GZ	110	300	Leaf	9	12.8	0.15
*P*. *persica* (225 Mb)	PXL^c^	11.1	21	Leaf	23	3.74	0.52
				Root^d^	13	29.8	4.06
	HY2	14	25	Leaf	16	6.19	0.62
				Petal	13	11.31	1.13
	NJAU1	15.9	30	Leaf	26	6.46	0.56
	NJAU2	37.6	50	Leaf	8	6.25	0.40
	Maoping	12.8	40	Leaf	16	3.56	0.26
	DHQ1	3.1	2	Leaf	75	1.97	2.54
*P*. *mume* (220 Mb)	MHG1	17.5	20	Leaf	25	12.9	2.17
				Root^d^	32	25.4	4.82
	MHG2	10.2	8	Leaf	33	5.7	2.38
*S*. *suchowensis* (480 Mb)	YAF1	-	1	Leaf	19	1.26	2.58
				Root^d^	21	2.86	6.60
*B*. *distachyon* (272 Mb)	WD2	-	1	Leaf	29	3.17	6.13
				Root^d^	8	4.75	8.97
				Lemma	7	2.57	4.97
*Fragaria vesca* (210 Mb)	FH1	-	1	Leaf	45	1.93	6.37
				Stems^e^	4	4.75	15.78
*Arabidopsis thaliana* (119 Mb)	Col17+Col24	-	1	Leaf	64	0.69	4.35
*Oryza sativa* (373 Mb)	KA1+PA1+ DG1+NIPB	-	1	Leaf (Tiller)^f^	29	4.79	9.01
				Leaf (Callus)	13	194.8	287.1

^a^Mutation rate per bp per year corrected for accessible genome regions.

^b^The age of G1 was estimated to be at least 600 years old by comparing with another about 900-year-old peach tree whose diameter is about 240 cm, and this tree is only a few kilometers away from G1.

^c^The age of the PXL peach tree was estimated to be about 21 years old through counting its annual ring in 2016.

^d^These four samples contain both leaves from different trunk branches and roots from different underground root branches; in samples of tree roots, DNA was extracted from the phloem of the root.

^e^In these strawberry samples, four runner regions in the same vine were chosen for sequencing (see Fig 3B for details).

^f^Tiller samples from culture-derived plants were not included.

We find that there are more mutations per sample in roots than in leaves in the perennials. The mean mutations per root and per leaf sample are 29.8 versus 3.74 in peach (Brunner-Munzel [BM] test, *P* < 2.2 × 10^−16^) and 25.4 versus 12.9 in plum, respectively (BM test, *P* = 3.8 × 10^−5^). This contrast was also evident in perennial shrub willow (2.24 in root versus 1.05 in leaf, BM test, *P* = 0.01), which has exactly grouped roots and leaves from the same cuttings (S2 Fig and S1 Table). However, although an absolutely higher number was also observed in the annual *B*. *distachyon* (4.75 in root versus 3.17 in leaf), the ratio is more modest, and indeed the difference is not significant (BM test, *P* = 0.48). It is notable that the ratio is most extreme in one of the two long-lived species (peach), near parity for the annual, and intermediate for the other perennials (relatively short-lived shrub willow and plum).

**Fig 1:**
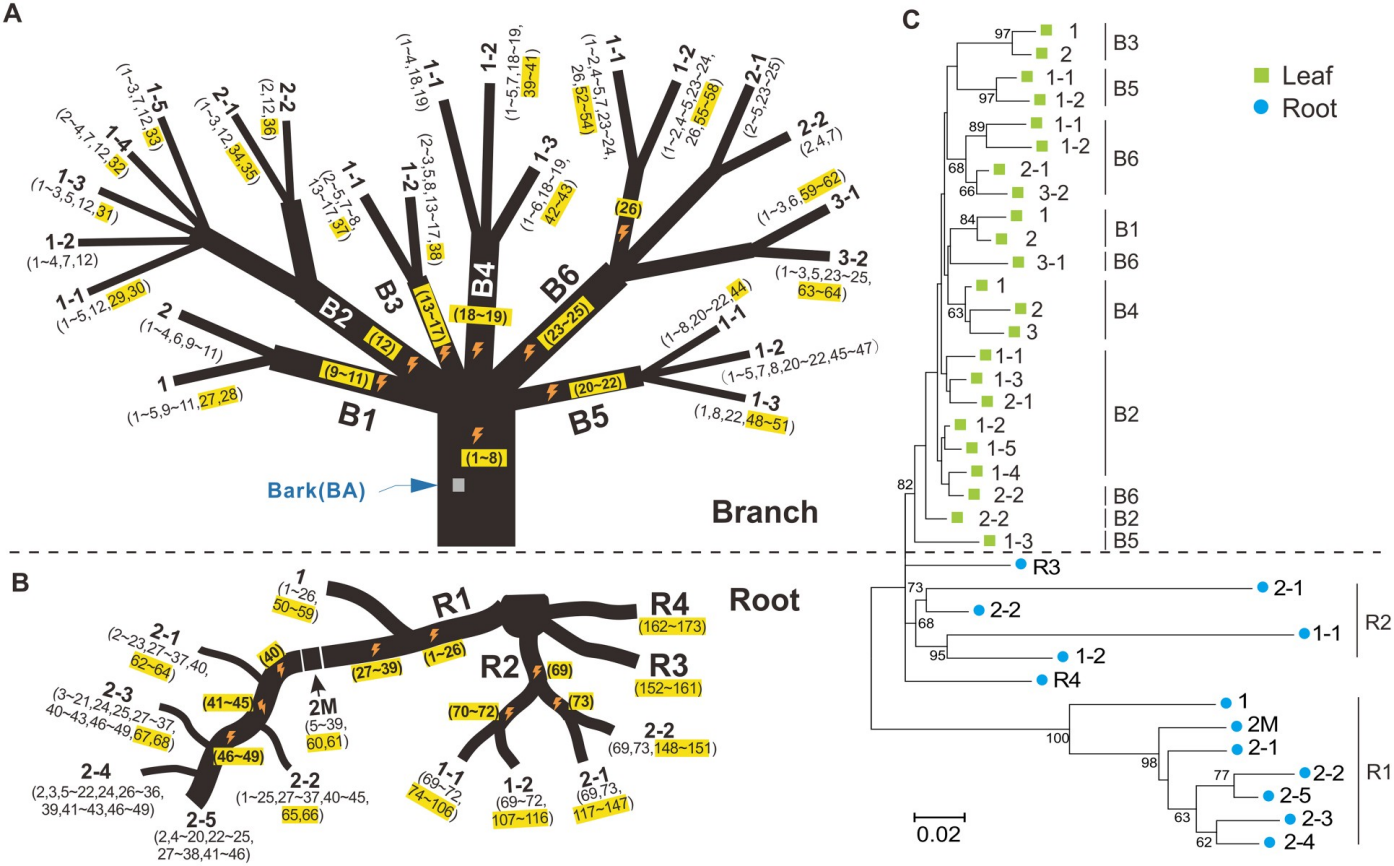
Pattern of somatic mutations in the branch leaves and roots of the peach tree PXL. (A) Somatic mutations identified in leaves. Each mutation is represented by a number (e.g., 1, 2, etc.) in parentheses. The first ontogenetic presence of a mutation is highlighted in yellow. In some instances, a mutation is unique to a terminal branch. If not, the orange lightning symbol represents the putative ontogenetic position of a mutation based on the shared pattern of this mutation. All branch dimensions are not scaled to the real size. (B) Somatic mutations identified in roots. (C) Ontogenetic trees constructed using all mutations identified in both branches (green) and roots (blue). Only bootstrap values over 50% are shown (neighbor-joining method with 1,000 replicates bootstrap test).

A visual way to observe the root—shoot difference is by observation of the topology of an ontogenetic tree (like a phylogenetic tree but reflecting mutations through development) constructed from all the leaf and root mutations of peach tree PXL. This displays the very evident differences of mutation patterns between the shoot apical meristems (SAMs) and root apical meristems (RAMs) (Fig 1), with root having very long “branch” lengths, consistent with very different mutation rates per unit time from the two tissues.

### A low proportion of transmissible mutations in annuals compared to perennials are somatic in origin

In the prior Results section, we found a general trend for relatively low stem mutation rates (per unit time) in perennial species but not in an annual. Might there be a reason that annuals and perennials are different in this regard? One possibility is that, as in animals, a short-lived species has less interest in restraining the mutation rate of all somatic tissues [3,4]. However, unlike soma in animals, stem mutations are potentially transmissible; thus, we might in addition predict that annuals should have a higher stem rate (and a stem—root relative rate near parity) if they also transmit relatively few premeiotic mutations. Consistent with this possibility, theoretical models predict that the contribution of somatic (pregametic) mutation could outweigh that of gametic mutation, especially in modular plants with small populations [14], such as long-lived trees.

Prior data on the rate of evolution of perennials and annuals are undecisive on this issue, as they do not assess the relationship between intra-organism mutation accumulation and the relative transmissibility of mutations. Whereas the per-generation mutation rate in long-lived perennials could be as high as 25 times as that in short-lived annuals [24], on the per-year scale, the long-lived perennials apparently evolved slower than short-lived annuals, as suggested by the generation-time hypothesis [25,26]. These data do not address the ratio of mutation accumulation to mutation transmission.

When considering transmissibility of mutations we can consider two metrics. In both, we estimate the number of mutations that are premeiotic in the parent but transmitted to progeny (*N*_t_). We can then consider this in proportion to the number of mutations observed in either the offspring (*N*_o_) or the parent (*N*_p_).

**Fig 2:**
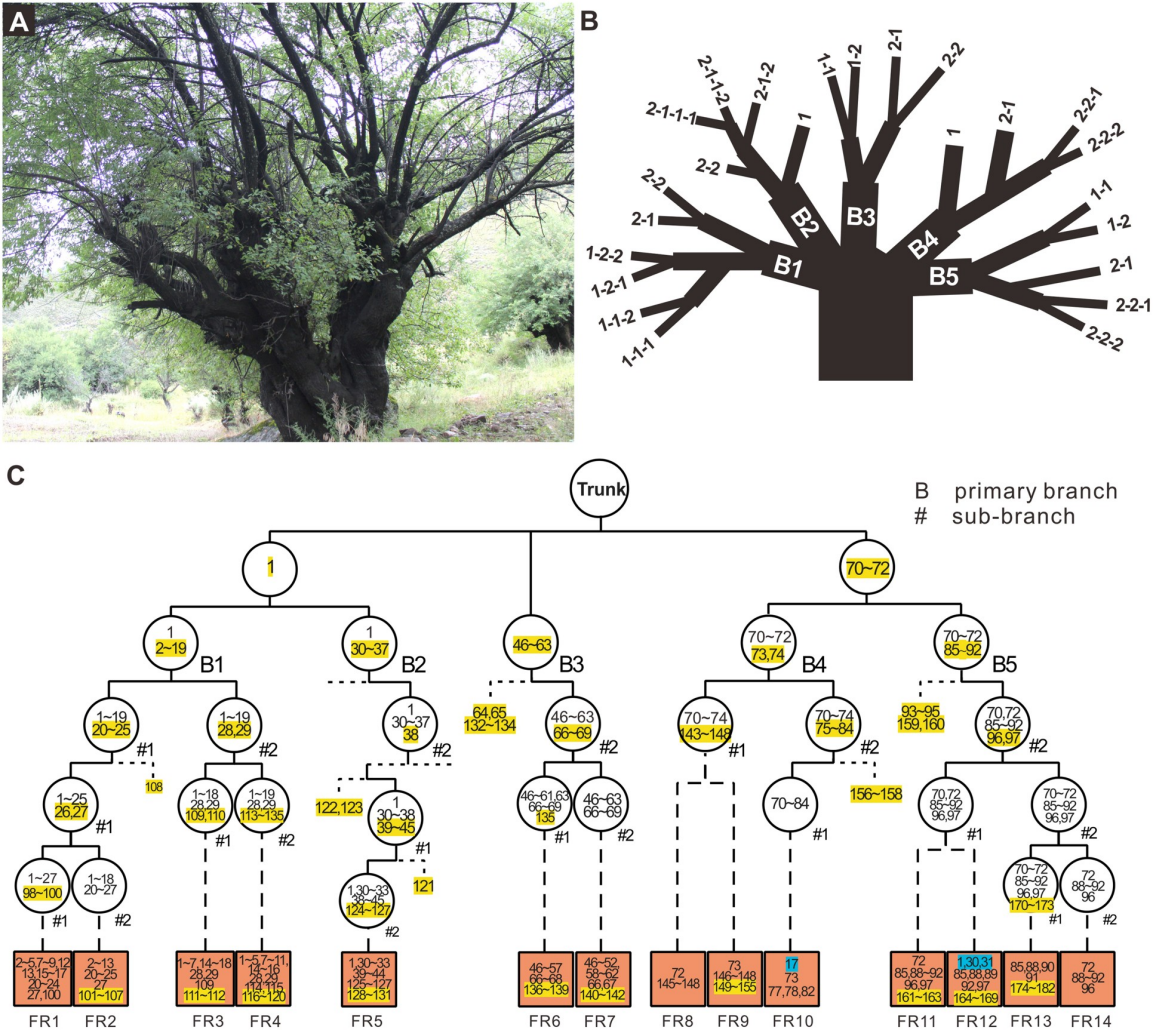
Distribution of somatic mutations and the mutations of meiotic progeny in an old peach tree. (A) Photo of the GL2 tree at Nyingchi in Tibet. (B) Sampled leaves in the terminal branches of the tree. (C) The history of the mutations. All mutations including somatic mutations from the tree and the mutations identified in 14 meiotic progeny are numbered. The yellow highlighted numbers represent the first occurrence of a de novo mutation in the tree’s history. The numbers without highlight represent the derived mutations from the previous level of a branch. Each circle represents the branching node where new mutations arise, and the brown box represents the progeny sampled from this branch. Branches with no fruit sampled are not shown (dashed lines beside circles) for better visualization. The unhighlighted and highlighted numbers in the brown box stand for the inherited somatic mutations from GL2 and the de novo mutations, identified specifically from the meiotic offspring, respectively. The blue highlighted mutations in the FR9 brown box are supposed to be inherited from the branch of B4 via outcrossing.

We start by considering the first ratio (*N*_t_/*N*_o_) in a perennial. To this end, 14 fruits from the tree GL2 (Fig 2A–2C) were harvested and germinated. The leaf DNA was extracted from these seedlings and sequenced. Based on the sequenced genotypes, 10 seedlings were self-pollinated products, and one was an outcome of putative inner cross between branches B2 and B5. Both mitotic (113) and “not premeiotic” mutations (47) can be unambiguously identified from the 11 self-pollinated products (Table 2), indicating that the majority of mutations observed in the offspring (71% and 66% in younger tree GZ; Table 2 and S2 Table) are derived from premeiotic mutations in peach trees. Note that the “not premeiotic” mutations are defined as all mutations from the meiotic progeny that are specific to meiotic progeny. Some of them may be generated by mitosis just before meiosis, and some may be from early development of the progeny. The other 3 seedlings were outcrossed products between GL2 and different peach trees, in which only the transmitted mitotic mutations can be easily determined. Further tests revealed an average of 3.86 premeiotic mutations per seed in the 21 fruits of the plum tree MHG1 (S3 Table), indicating that premeiotic mutations are a major source of genetic variation in perennial species.

**Table 2:** Mutations identified in 14 meiotic progeny of wild peach GL2.

Fruit ID	Inherited somatic mutations	Specific mutations in the progeny^a^	Proportion of the inherited mutations^b^
FR1^c^	19	−	NA
FR2	18	7	18/25 (72%)
FR3	15	2	15/17 (88%)
FR4	17	5	17/22 (77%)
FR5	14	4	14/18 (78%)
FR6	15	4	15/19 (79%)
FR7	14	3	14/17 (82%)
FR8^c^	5	−	NA
FR9	4	7	4/11 (36%)
FR10^c^	5	−	NA
FR11	9	3	9/12 (75%)
FR12^d^	8	6	8/14 (57%)
FR13	4	9	4/13 (31%)
FR14	7	0	7/7 (100%)
**Mean**	11.0	4.6	125/175 (71%)

^a^The specific mutations in a progeny could occur during meiosis or mitosis related specifically to this progeny.

^b^The proportion represents “premeiotic mutations that get transmitted/number of total mutations in progeny, *N*_t_/*N*_o_.”

^c^These three individuals are supposed to be progeny from outcrossing with another *P*. *mira* tree. In those cases, it is difficult to identify the de novo specific mutations because of the pollen derived from different trees. However, the inherited mitotic mutations are easy to identify.

^d^This sample is a putative inner cross between branches B2 and B5, and the other 10 plants are self-pollinated products. In those genomes, it is easy to identify both the inherited somatic mutations and the de novo specific mutations in each progeny.

Abbreviation: NA, not applicable.

The *N*_t_/*N*_o_ ratio is much lower in annuals. Sixteen whole-genome-sequenced progeny of *Brachypodium* sample WD2 indicated that only 24% of mutations in these seedlings are derived from premeiotic somatic mutations, with 0.69 premeiotic somatic mutations on average in any given progeny (S4 Table). This proportion is much lower than that seen in trees (for GL2 it is 71% [Table 2], for GZ 66% [S2 Table], higher than 24% in the WD2 [S4 Table], χ^2^ = 32.835, *d*.*f*. = 1, *P* = 1.003 × 10^−8^). Note that for all cases, both the annuals and perennials, the progeny DNA was sampled after approximately 1 month of growth.

**Fig 3:**
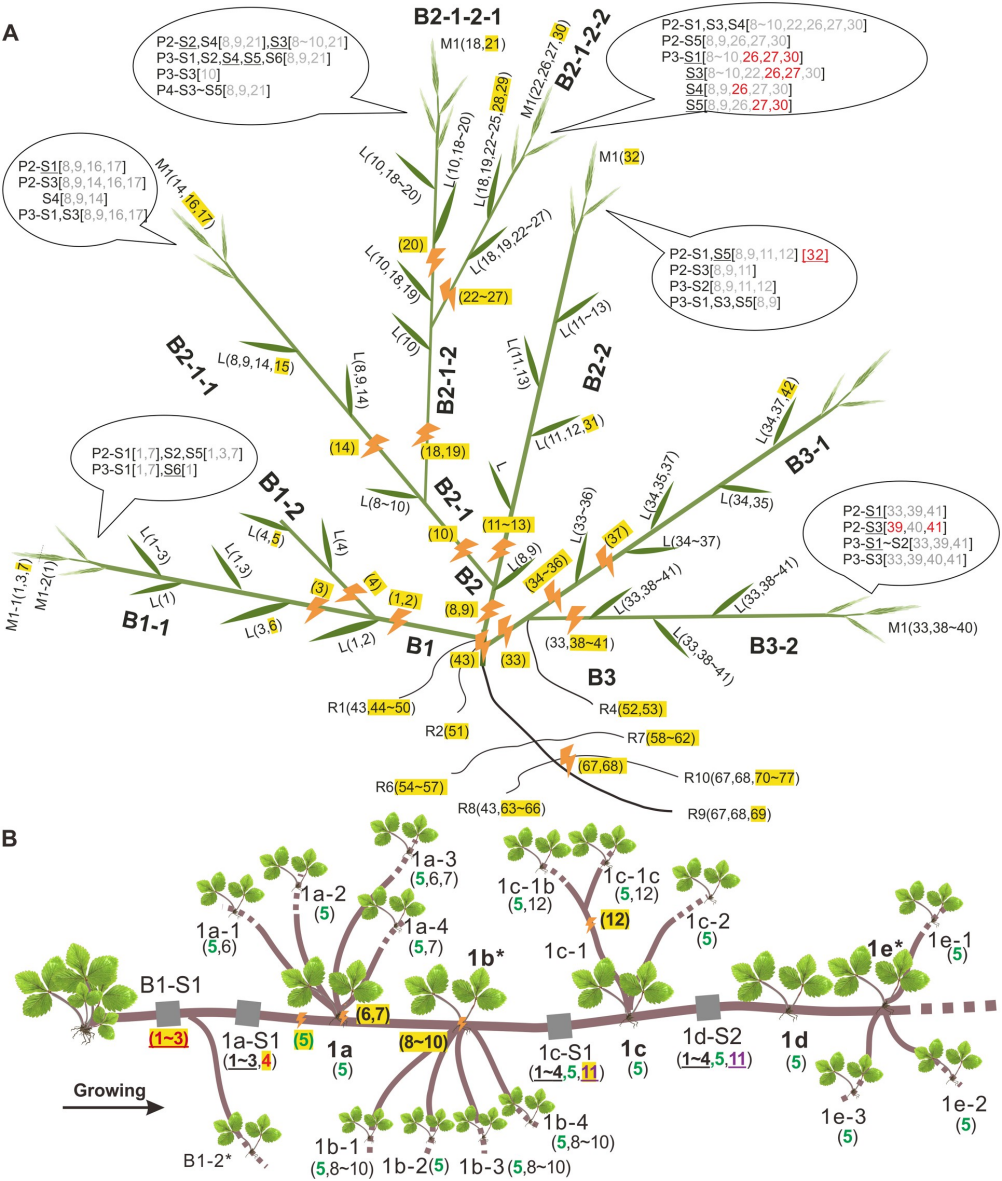
Patterns of somatic mutations in *B*. *distachyon* WD2 and woodland strawberry FH1 runners. All mutations are numbered or marked in the same manner as those in Figs 1 and 2. (A) For each branch of *B*. *distachyon*, multiple leaves were sampled (marked with abbreviation “L”). The glume/lemma samples (“M”) were carefully collected from one spikelet. For branch B1-1, two glume samples were obtained, with M1-1 from the uppermost of the spikelet and M1-2 from the lowermost part. For other branches, only one glume sample was collected. Root samples (named as R1–R10) were also collected and carefully cleaned for DNA extraction. Seeds (“S”) from another spikelet (“P”) of the same panicle were grown into seedlings to get DNA. For example, two panicles P2 and P3 were collected from branch B1-1, and three seeds (S1, S2, S5) from P2 were used for PCR verification. Fourteen of them, which were also sent for WGS, are marked with underlines. Mutations designed for testing of inheritance are shown in square brackets behind each progeny. A mutation confirmed to be inherited by PCR and Sanger sequencing is marked in red and otherwise in gray. One inherited mutation (32) confirmed by WGS is marked by red and underlined. (B) For woodland strawberry, both leaves (e.g., 1b-1 and 1e-3; for each plantlet, or daughter plant, only a single leaf was sampled) and runner samples (1a-S1, 1c-S1, etc.; gray, each about 15 cm in length) were sequenced. The mutation 5 is colored green to show its wide distribution in all samples sequenced. Samples marked with asterisks (1b, 1e, etc.) were not sequenced because no qualified DNA was obtained. Note that the weight of the approximately 15 cm of runner was about 100–150 mg, which was slightly higher than that of leaf (a single leaf sampled per daughter plant roughly weighed 70–100 mg). The runner DNA is harder to extract, so the total DNA mass from the runner would be a little less than from leaf. However, as usually only around 200 ng of DNA was used in library preparation, the mass of DNA that actually was sequenced should be similar between leaf and runner. WGS, whole-genome sequencing.

The alternative ratio, the proportion of premeiotic mutations that get transmitted/total premeiotic mutations, *N*_t_/*N*_p_, is also lower in annuals. In total, we did 317 PCRs for 49 mutations in a total of 115 seedling samples in *Arabidopsis*, rice, and *Brachypodium* (S3 and S4 Figs, Fig 3A). We found 1.72% (1/58 per seed), 3.0% (3/100 per seed), and 6.29% (10/159 per seed) of premeiotic mutations to be transmissible, respectively (*N*_t_/*N*_p_). This proportion of transmissible mutations is significantly lower (Pearson’s χ^2^ test with Yates continuity correction, χ^2^ = 187.53, *d*.*f*. = 1, *P* < 2.2 × 10^−16^) than that observed in trees (approximately 51.6% overall, with 154/305 = 50.5% per progeny in GL2, 61/124 = 49.2% per progeny in GZ, and 17/21 per progeny = 81.0% in Maoping). Note the plum MHG1 (84/319 = 26.3% mutations per progeny transmitted) was not included in this comparison, as its progeny are from outcrossing whereas all others are selfing, which would make the comparison unfair because a mutation in selfing progeny has a greater chance of being transmitted. It is then all the more striking that the absolute rate in this outbred individual is higher than in the selfing annuals. The high number of somatic mutations in annuals is similarly reflected in the large number (3.32 per leaf) in rice (S4 and S5 Figs) with low transmissibility.

The low transmission in annuals is similarly reflected in the spatial location of the few mutations that are transmitted, these typically arising in the vicinity of the sites of gametogenesis. For example, the *Brachypodium* sample WD2 has eight branches derived from three major branches, from which 29 leaf samples and seven glume/lemma samples (each from a spikelet and each branch usually growing three spikelets in general) were sampled (Fig 3A). In addition, 42 seeds were collected from different spikelets within the same branch as the glume samples. In the 36 leaf and glume samples, 77 novel mutations were detected. Among the 77 mutations, 22 were selected for further PCR and Sanger sequencing to see if they were transmissible. Just under one-quarter (5/22 = 23%) of them were confirmed to be present in any of the five seeds. Further whole-genome sequencing of 16 progeny suggested this rate was actually lower (8%), with only six mutations (five were those confirmed by PCR) present in any of six seeds among 40 mutations within the parental branches bearing those seeds (Fig 3A). Five out of the six mutations are present in the seven glumes that have been sampled and sequenced. This indicates that mutations physically closer to the site of gametogenesis (i.e., in glumes) have a greater chance of being transmissible (χ^2^ test with Yates correction, χ^2^ = 16.9, *d*.*f*. = 1, *P* = 3.86 × 10^−5^). As an independent assessment of quality control, we note that the transmitted mutations identified in the sequenced genomes exactly match the PCR-verified results.

We conclude that annuals transmit proportionally fewer of their premeiotic mutations than do perennials and that a lower proportion of mutations reported in the progeny are premeiotic in origin. This in turn could explain, from an adaptive model, why the root—shoot mutation rate ratio is high in perennials but not in annuals: if premeiotic mutations have little chance of transmission and there is no possible accumulation year on year (i.e., in annuals), then relatively little is to be gained by reducing the shoot mutation rate.

### Woodland strawberry as the exception that proves the rule

All the data presented here support an adaptive framework well. In perennials, the shoot mutation rate is relatively constrained because they transmit shoot-accumulated mutations, whereas the shoot mutation rate is relatively unconstrained in annuals because they transmit relatively few mutations and the plant will shortly be dead. In this context, one observation appears, prima facie, to be an exception and counter to the selectionist model. In woodland strawberry, an initial plant sends out runners that can occasionally produce lateral buds that initiate new plants with shoots, leaves, and fruit (Fig 3B). Thus, every gamete in a runner-propagated mature plant has an ontogenetic cell lineage history that runs back through the runner to the parent plant and thence to the initial seed. We might therefore expect that runner, as the progenitor of all plants and hence of all seeds, to have an especially low mutation rate. However, we find that there are 4.75 mutations accumulated per runner versus 1.93 per leaf (BM test, *P* = 8.2 × 10^−7^; Table 1). Counting each mutation once, we find 0.67 mutations per daughter plant, and 2.33 mutations per node for runners. Why is the runner rate higher?

The example of nontransmissibility of somatic mutations in annuals suggests a related explanation. What if mutations that occur in runners are for the most part not passed on to the lateral buds, as the cell lineage permitted to develop into lateral buds is spatially restricted? Were this the case, most cells in the runner would be more like root in having no ontogenetic future in gametes and hence would be under relatively relaxed selection. By contrast, cells of the shoot of the plant would still have a potential future in gametes. Does then the runner contain an effective germline?

Analysis of mutation accumulation patterns (Fig 3B) provides strong support for the possibility of two separate cell lineages, one that is ontogenetically restricted to the runner and one that is not. Because we know which was the first plant, we know both spatially and temporally where all the new mutations initially occurred and when they are subsequently found. We find a pattern in which mutations found in the runners are normally restricted to the runner, but with one exception. Consider the first three mutations (numbered 1 to 3 Fig 3B). These appeared in runner site B1-S1 and can be detected in the subsequent runner sites of 1a-S1, 1c-S1, and 1d-S2 but could not be detected in the subsequent shoots and leaves produced from the lateral buds (lateral buds 1a to 1e, resultant shoot/leaves 1a-1, 1a-2, etc.). Similar ontogenetic restriction was also found for all subsequent runner mutations (4 and 11), bar one.

The one exception is mutation 5, which occurs prior to stem/runner 1a but is henceforth seen everywhere: in all progeny runners and in the products of the five lateral buds (1a to 1e). It is notable that at positions 1c-S1 and 1d-S1 in the runner, we find all of the runner mutations (1–5 and 11), but in the lateral bud progeny (1c, 1d, 1e) mutation 5 alone is seen (excepting mutations that arose in the shoots/leaves, e.g., mutation 12).

Providing a statistical test for the exceptionalism of mutation 5 requires a few assumptions. But let us suppose that each lateral bud has, as observed, only one mutation seen in the runner from which it is derived (this could be owing to a small initial cell population founding a lateral bud). At 1a, there are five runner mutations, and we can then suppose that one (and only one) of these was transmitted to descendent shoots and leaves. We attach no probability to this first selected mutation being number 5, as this is only relevant post hoc. Instead, we ask what the probability is that for the subsequent plants/lateral buds the same one mutation (whichever it is) is selected at random, this being the null. At 1b, the runner has mutations 1 to 5, so the probability of any prespecified mutation being the mutation in the lateral bud is 1/5. At 1c to 1e, these five mutations are joined by mutation 11. Thus for each of these, again, assuming one successful mutation, the probability that the prespecified mutation is in the lateral bud is 1/6. Thus, the probability of the initially successful mutation (in our case, mutation 5) alone being selected at each lateral bud is 1/5 × (1/6)^3^ = 0.00093. This provides strong reason to reject the null of random cell selection in the production of lateral buds and, conversely, supports the possibility that in runner, there is a segregated germline.

Although highly significant, the above calculation comes with numerous caveats. We assume only one mutation can be transmitted to lateral buds. However, a correct null of random cell selection (rather than random mutation selection) would make it even less likely that all subsequent lateral buds would have the same prespecified mutation, as nontransmission must be an alternative part of parameter space under such a null, there being no reason to suppose that every cell has a mutation. Inclusion of a nontransmission possibility thus renders the likelihood of the same mutation being transmitted to the lateral bud every time even less likely. However, we do not know the number of progenitor cells in the lateral bud or the proportion of cells with at least one mutation and so cannot specify this null correctly. Furthermore, we assume all mutations to have occurred in different cells and so are themselves independent in any model of random cell selection. This need not be true.

Despite the above caveats, it is most parsimonious to suppose that one mutation (5) uniquely occurred in a germline lineage within the runner and that only mutations in this cell lineage make up the shoot derived from lateral buds. Other lineages may make up further runners (1–4, 11) but are restricted from lateral buds and hence are not in shoots, leaves, and gametes. Because of such a restriction, a relatively unconstrained mutation rate can be expected. Thus, the one prima facie exception may be the exception that proves the rule.

### A greater load of mutations in petal than in leaf

The stem—root difference is consistent with a selectionist view of mutation rate variation within a plant. The same model could also predict that longer-lived terminal tissues might have lower mutation rates than shorter-lived tissues, just as a soma in short-lived species has a higher rate than a soma in longer-lived species. The leaf—petal difference is here a potentially informative test. Petals comes from the second whorl formed by the floral meristems [27] and have a similar cell division profile to leaves [28]. As the floral meristem shares a similar organization with SAM, it has been suggested that flowers and shoots are homologous structures, with floral organs being viewed as modified leaves [27]. But differences do exist; for example, unlike the stem cell fate in SAM, which is indeterminate (i.e., not determined by its cell lineage but by its position) and grows indefinitely, the stem cells in floral meristem are determinate and will cease growth upon the formation of four whorls [27].

We consider two sampling strategies to examine the leaf—petal difference. First, using whole tissues, we observe that peach petals have a higher rate than leaf samples (11.31 versus 6.19; BM test, *P* = 0.007). Second, we consider sampling of tissue by using microholes. To this end, two peach leaves and four petals were sampled from different branches of the tree HY1. In two leaves, 16 tiny holes, each containing about 1,000 cells, were punched (S6 Fig and S5 Table). After amplifying and sequencing those samples, 59 mutations were identified, with an average of 3.69 mutations per hole sample in leaves, much lower than 1,567 mutations per hole in petals.

Although this latter result agrees qualitatively with the prior result using whole peach leaf and petal samples, the ratio is clearly much higher when using the microhole methodology. The mutation number seen in petals is so high we must suspect a technical artifact. Our method involves each petal sample being compared to all other petal and leaf samples (i.e., between different tissues) from the same tree, and the mutations being called are those unique to a single hole sample and not in any other leaves or petals. This is potentially prone to false positives, as it requires few consistency checks and could be liable to sequencing artifacts introduced during amplification. We can be confident that the numbers are not sequencing errors, as 39 mutations that we can retest via Sanger sequencing 36 (92%) are verifiable. However, we may be doing little more than confirming amplification artifacts. To be confident of a qualitative difference between leaf and petal, we therefore also ask about mutations that are shared between different microholes but are specific to a single flower/leaf. Such mutations are unlikely to be amplification or sequencing artifacts. We observed 73 and 16 mutations in two flowers (the four petals belong to two flowers from two branches) that were present in at least two microhole samples (both supported by at least five reads for the mutation allele). This contrasts with 4 and 2 shared mutations in two leaf samples from two branches (one-sided comparison of Poisson rates, *P* = 0.000974). Although this does not resolve the cause of the remarkably high mutation number called singly in microholes of petals, it reinforces the conclusion that petal has more mutation accumulation than leaf and, as such, is consistent with highly relaxed selection in very short-lived petals.

### No evidence of excessive between-branch heterogeneity in mutation rate

We have provided evidence that different plant tissues have different rates of mutation accumulation, the variation being consistent with an adaptive optimal allocation model. A further prediction is that the variation should be deterministic and hence that between biological replicates there should be no more heterogeneity than expected under a null of equal rates.

We address this issue by asking whether different branches also differ in their mutation rates. A key problem in any such analysis is controlling for heterogeneity in the number of new mutations held on a branch that results from something as trivial as different ages of branches. To circumvent this, we consider 75 terminal branches on a young peach tree (DHQ1) in which we can be confident that all the branches sampled are of approximately the same age. We then consider the number of mutations that are unique to any given branch. If the null model is correct, the distribution of these numbers should be a Poisson function, and hence the dispersion (= Variance/Mean) should be no different from unity. We find the dispersion is D = 1.031. Significance we tested via simulation (10,000 replications), deriving a mean D in simulants of 1.0 ± 0.162 (SD). The observed dispersion is thus no higher than expected by chance (from simulation, *P* = 0.454). Although then branch-specific mutations can be found (each branch has on average 0.45 branch-specific mutations), we see no evidence for between-branch heterogeneity in rates.

### Tissue culturing is associated with a high mutation rate

The in vivo evidence is broadly consistent with selectionist models in which we expect a lower mutation rate in cells in which any mutation has a larger potential future impact (longer-lived terminal tissues or potential germline tissue) and the variation observed is deterministic. But might there also be variation that is nonadaptive and better explained by mutational fragility? We address this by comparing plants grown under very different conditions but over the same time span. The artificial condition is tissue culture, which we compare with the same plant grown in the wild.

We considered a 1.1-mg callus derived from a single rice seed. This was cultured to 657.3 mg (about 10 cell divisions) and then divided into five groups with 10 seedlings regenerated from each of them (S7 Fig). When the leaves were sequenced, the mutations specifically generated during culturing can be identified. This results in an average of 357 mutations for each seedling (each regenerated plant was grown for approximately 2–3 months before sampling), which is approximately 47-fold higher than the number of mutations accumulated among different tillers in the same plant (S6 Table and S7 Fig), even though the wild-grown plants are possibly older (grown for about 3–4 months before sampling), indicating a high rate of mutation per unit time in the callus.

### No evidence for intra-organismic selection

We have presented evidence for differences in rates of mutation accumulation between different tissues in the same plant that we have postulated to be owing to differences in the mutation rate. However, an alternative possibility is that different tissues have the same mutation rate but differential degrees of purifying selection. Although this is unlikely to explain more-extreme differences, this has been a potential issue in the debate as to whether plants have a mutationally protected germline: prior data suggesting this [11,12,29] have been argued to alternatively be explained by purifying selection removing mutations, not by mutation not generating them [16,30].

Here then, we ask whether intra-organismic purifying selection is an important problem (nota bene: we do not attempt to directly ask if any putative germline has a low mutation rate). We adopt two approaches. First, we ask whether the rate of mutational accumulation decreases as a function of age, taking advantage of our ability to determine, given the branching structure of a plant, when any given mutation arose. Second, we compare the transmissibility of harmful (nonsynonymous) and less harmful mutations. In neither case do we detect a signal of purifying selection. We thus presume that our measures of mutation accumulation are not profoundly confounded by intra-organismic selection. In addition, this supports the evidence that plants might have an effective germline [11,12,29,37]—i.e., an early segregating and slowly dividing germline that accumulates few premeiotic mutations—as the low number of germline mutations cannot be easily explained by purifying selection.

#### 1. Somatic mutations accumulate at a constant rate

If purifying selection is an issue, we might expect a decline in the rate of mutation accumulation year on year, under the premise that selection takes time to weed out mutations or to let slightly fitter lineages dominate, much as very recently diverged species have very high Ka/Ks ratios [31]. This test is, however, imperfect because it assumes that the underlying mutation rate is not an accelerating function with respect to age, which could mask an apparent reduction caused by purifying selection. Accumulation of somatic mutations is, however, thought to contribute to the ageing of plants [22,24,32], and such accumulation could possibly result in more mutations that themselves increase the mutation rate, thereby causing an accelerating function with age. Similarly, classical theories of senescence could predict that mutation rates might increase with increasing age, thus leading to higher rates of mutations in older plants. Nonetheless, with this caveat, to survey the yearly variation of somatic mutation rates, we sequenced the leaves of a young tree so as to estimate the number of new mutations per leaf per diploid genome per year and of accumulated mutations per leaf per genome.

**Fig 4:**
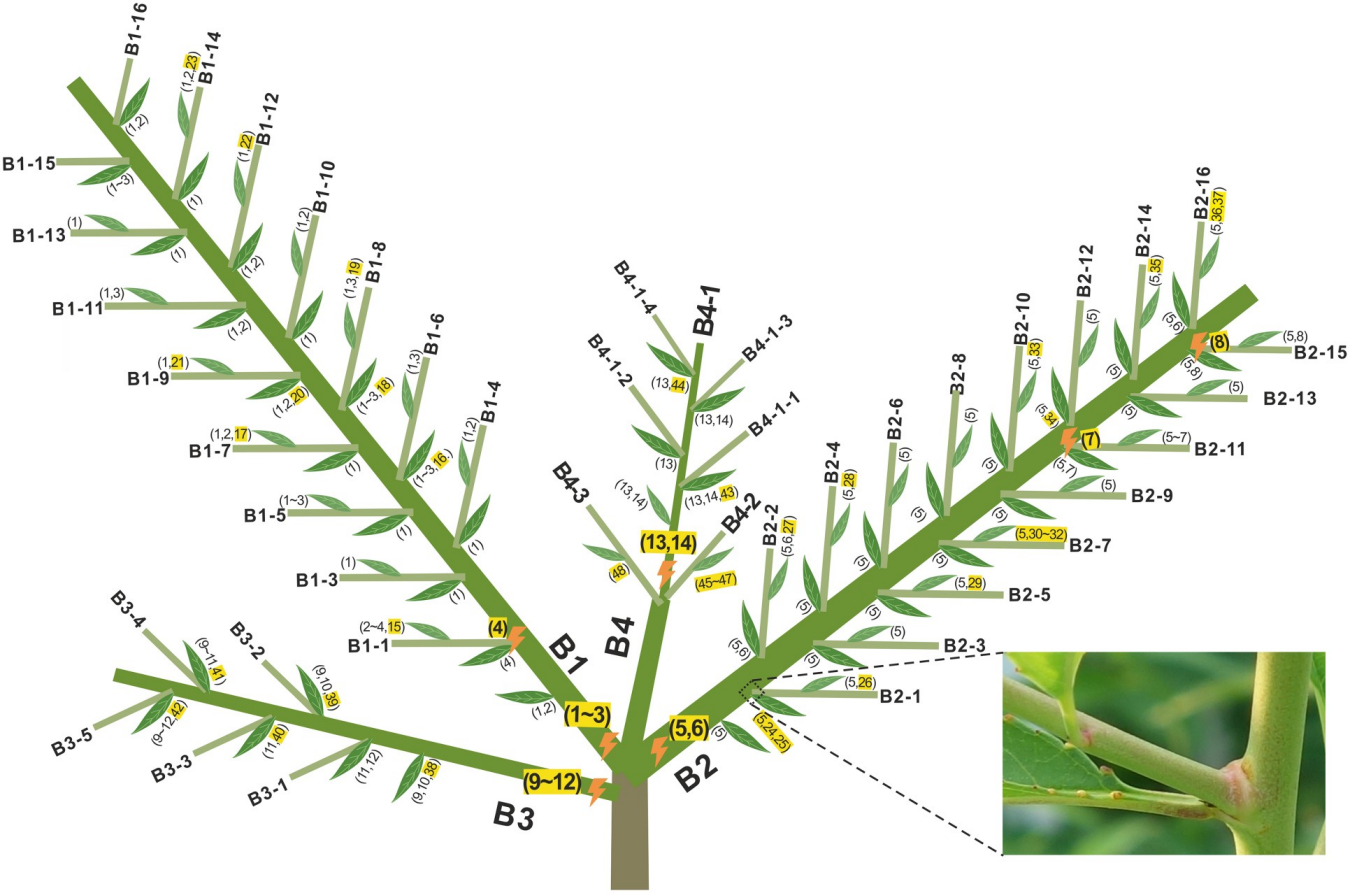
Somatic mutations in a 2-year-old peach tree DHQ1. This tree was from a seed germinated after gibberellin treatment from May to June 2015 and grown outdoor on the campus. The leaves were sampled from the sapling on July 24, 2016. Seventy-five sequenced leaf samples include 43 leaves from the base branch (shown in rectangle), and the others are from small branches.

We harvested a fruit from a peach tree and germinated it in June 2015. This seedling (DHQ1) had four branches in 2015 (B1-B4 in Fig 4) and more subbranches in 2016. Seventy-five leaf samples collected in September of 2016 were sequenced. For these, we could identify new mutations and can be confident that they must have occurred in 2015 if the mutation was seen in different leaves on the same branch. The remainder we presume to have occurred in 2016. In total, 48 de novo mutations were identified (colored yellow in Fig 4). Out of these mutations, 11 mutations were shared by leaves in a branch and so must have occurred in 2015. From this, we can estimate the number of mutations that accumulated in 2015 per leaf. For example, the frequencies of mutations 1, 2, and 3 in B1 are 27/29, 14/29, and 8/29 in 29 leaves, respectively, which results in 1.69 mutations or 1.44 on average in B1-B4 accumulated in 2015. Similarly, mutations per leaf in 2016 can be calculated as 0.53. The sum of accumulated mutations per leaf over 2 years is 1.97 (Table 1). The distribution of novel mutations, particularly the 11 mutations that occurred in 2015, indicates that the mutation rate detected for the second year may be only about one-third of that for the first year (the null expectation is that the same number of mutations accumulated in all sampled leaves each year, observed = 108 mutations accumulated in 2015 and 40 accumulated in 2016, χ^2^ with Yates correction = 30.3, *P* = 3.64 × 10^−8^). This is not consistent with the possibility of an accelerating function with age, the form that would mask apparent germline purifying selection.

Is the trend that we see for a young plant for fewer mutations later on than early on replicated over broader time spans and in mature plants, or might this reflect a burst of mutations early on? We find no evidence for anything other than a constant rate of mutation accumulation. The net accumulated mutations (Table 1) range from 1.97 to 23.9 per leaf sample in four old wild peach trees (*P*. *mira*; G1, G2, GL2, and GZ; about 200–600 years old [33]) and six young peach trees (2–50 years old in *P*. *persica*). By using the diameter of a tree as a proxy for its age, a positive correlation is obtained between age and base mutations accumulated (*y* = 0.134*x* + 4.195; *R*^2^ = 0.838 and *P* < 0.001). There is a suggestion of an early accelerating function (consistent with the above observation), but the intercept of the linear plot is not significantly different from zero (*P* = 0.09), arguing against this being a strong effect. There is no suggestion of an accelerating function with age, and indeed when we consider the difference in the number of accumulated mutations per leaf per unit difference in trunk diameter as a function of trunk size, there is no significant correlation, consistent with rate constancy (Spearman rho = −0.4, *P* = 0.29). An accelerating or decelerating function would predict a positive or negative correlation, respectively.

A problem with the above method is that we employ trunk diameter as a proxy for age. By sampling young branches (a branch only grown for about 1–5 years), we can examine the accumulation rate with greater certainty over age but over a shorter time span. When leaves are sampled from any of those terminal branches, the mutations accumulated in a certain year can be distinctly identified. From four peach trees for which we documented the age of several of their branches (G1, G2, PXL, and NJAU1 in Table 1), we identified 1.64, 2.64, 3.76, 3.55, and 5.83 mutations in 1–5 years of branches, respectively. A positive correlation is present between the age (*x*) of a branch and the number of somatic mutations accumulated per leaf (*y*) (*y* = 0.929*x* + 0.698; *R*^2^ = 0.890 and *P* = 0.016). The function is again well modeled as a linear fit and the intercept not significantly different from zero (*P* = 0.35). The rate of mutation accumulation per year as a function of age is also not different from a null of constancy (Spearman rho = 0.4, *P* = 0.75) with a mean of 1.05 mutations per year. The constancy argues against purifying selection as a confound, except if the true mutation rate is an accelerating function with age, for which we find no support.

The profile of 29 mutations in young peach tree DHQ1 also sheds light on the cell processes leading to the development of a branch. We find no shared mutations between branches (nota bene: very early mutations shared between all branches would have been missed). This suggests that each branch is derived from a specific area of SAM. The shared mutations within a branch normally have different frequencies (e.g., 27/29, 14/29, and 8/29 in B1), which indicates that at least three or more cell divisions in a hierarchical arrangement are necessary for the development of a branch.

#### 2. No evidence that mutations of stronger effect are less transmissible

We can also ask whether our data are consistent with intra-organismic purifying selection by considering the transmissibility of nonsynonymous mutations and those less likely to be deleterious (synonymous, intronic, and intergenic). Under the null model that intra-organismic selection is a negligible force, we expect the profile of somatic mutations to be the same as the profile for mutations that were transmitted to the offspring. If selection on these timescales was important, then we might expect transmissible mutations to be enriched for the mutations less likely to be deleterious. We find no evidence for selection. Of all 132 somatic mutations identified in GL2, 14 are nonsynonymous, and 118 are less harmful. No significant difference is found between the transmissibility of nonsynonymous mutations and less harmful ones (observed 9 nonsynonymous mutations and 71 less harmful mutations are transmissible, expected 8.5 and 71.5, χ^2^ with Yates correction = 3.03 × 10^−5^, *P* = 0.996). We conclude that we find no evidence for within-individual purifying selection.

## Discussion

### Is mutation rate variation owing to selection?

We postulate that if selection is acting on the rate of mutation accumulation in plants, then stem should commonly have a lower rate than shoot; that highly ephemeral structures such as petals should have higher rates than ontogenetically related but longer-lived structures (i.e., leaves); and that mutation accumulation rates should be deterministic (i.e., no more between biological replicate variation than expected under a null). We find all to be upheld and, in addition, that the one exception (runner mutation rates in strawberry) may well be the exception that proves the rule, as it appears to have a segregated germline. The high mutation rate in tissue culture, however, suggests that mutation rate is quite easily altered by changes in local environment. Evidence that stress can cause increases in the mutation rate may well partially explain the callus result [34] and in turn suggests that we need to be cautious in our interpretation of between-tissue differences. The variation that we observe suggests that in plants, mutation accumulation rates are deterministically variable between different parts of the same plant. This does not argue in favor of either hypothesis (selection versus fragility), assuming that given tissues have consistent mutational microenvironments.

Although then some of the heterogeneity that we observe is predictable from an adaptive model (e.g., stems have lower rates than roots) and some predictable on anatomical grounds (different mutations in different branches), we cannot definitely rule out nonadaptive mutational fragility. We propose that in testing adaptive theories of intra-organismic mutation rate variation, the alternative hypothesis should be that heterogeneity reflects mutational fragility that is conditional on local context. The petal—leaf differences we see could, in principle, be consistent with either hypothesis. By contrast, evidence for variation in the shoot—root difference as a function of the proportion of shoot mutations that are transmissible argues against microenvironment and in favor of the adaptive hypothesis. This does not rule out the possibility that the root microenvironment is more mutagenic than the shoot environment, but any such effect cannot obviously explain why the root/shoot ratio varies with the proportion of shoot mutations that are transmissible. We conclude that prima facie, at least some of the mutation rate variation observed best fits a selectively driven model, whereas some is just consistent with such a model.

### Are differences owing to the number of cell divisions or the rate per cell division?

Regardless of whether the variation that we have observed is predictable in an adaptive framework, we can in addition ask whether the difference in mutation rates (leaf—petal, root—shoot) that we find reflects differences per cell division or differences in the number of cell divisions [35]. As far as adaptive theories are concerned, reducing either parameter would be an effective means to reduce the net rate of mutation accumulation.

The root—shoot differences we suggest probably reflect differences in rates per cell division. Our strategy required a mutation to be called when the majority of a leaf (or a root) shares a single given mutation. The requirement means that this mutation is most likely to be derived from a single or a few cell divisions, and this division must occur at a very early stage of leaf (or root) development. When defining a mutation with ≥5 reads in a total of 40× genome coverage (the maximal mutant reads is 20× in a diploid, assuming no bias), on average the mutation must be shared by ≥25% of cells in a sample. In other words, our sampling strategy will detect mutations that occur in one of a few early cell divisions for a leaf or root. Therefore, the mutations observed from such samples most probably reflect the mutation rates of SAMs or RAMs per cell division. Similarly, the high rate in the callus is probably best explained by an increased mutation rate per cell division. Thus, although the concentration in focus has been on strategies to minimize the number of cell divisions to protect the germline, the possibility of modification of the per-cell-division mutation rate, as seen in humans [5], should not be discounted.

The suggestion that the differences between shoot and root might reflect per-cell-division differences also accords with prior evidence suggesting that root and shoot have similar growth profiles. Estimation of the mitotic index, for example, indicates that the duration of mitotic cycle is roughly the same in shoots and roots [36]. This does not, however, take into account the finding that both SAM and RAM contain some cells that divide much slower than others [11,12]. This is likely to influence mutation accumulation because somatic mutation occurrence is correlated with the number of divisions [35], regardless of the per-cell-division rate.

### Do plants have a germline: Woodland strawberry as a useful model?

Prior work has suggested that plants might have an effective germline [11,12,29,37], which may be early segregating and slow dividing, thus accumulating few premeiotic mutations. We did not seek to test this hypothesis directly, but our lack of evidence for intra-organismic selection argues against the hypothesis that the reduced mutation rate observed by others may be more apparent than real.

Although we did not set out to test the germline hypothesis, our analysis of woodland strawberry strongly supports the possibility that runners in this species have two discrete cell lineages: one that can be propagated to future runners but not to lateral buds and one that can be propagated to all (i.e., a germline). The relatively high mutation rate in runners makes sense, if this is the case, as most of the runner is “root-like” in having no gametogenic future.

Our data, however, have little to say as to whether this germline is mutationally protected. There is no further mutation in runner that cosegregates (ontogenetically) with the germline one (mutation 5) that would be consistent with a low rate. However, after mutation 5 in runner, we see only one further mutation (mutation 11), all others occurring after lateral bud development. The chance that this new mutation would not be germline must be very high, even if there is no mutation rate difference. We suggest that this system would be valuable for further interrogation of the hypothesis of a mutationally protected germline and for mechanisms of cell lineage sequestration, not least because runner also makes a helpful control for the possibility that root might have a high mutation rate owing to its subterranean environment.

## Materials and methods

### Sample preparation

We collected a total of 22 plant individuals, including seven peach (*P*. *persica*) and four wild peach (*P*. *mira*) trees, two plum (*P*. *mume*) trees, one woodland strawberry (*F*. *vesca*), one shrub willow (*S*. *suchowensis*), four rice (*O*. *sativa*), one *B*. *distachyon*, and two *A*. *thaliana* individuals (S7 Table). The sampled individuals cover a life span range from several months to hundreds of years and three distinct genera. One of the peach trees was sampled in Maoping, Guizhou Province, China, and the others were from Nanjing, Jiangsu Province. The wild peach trees were sampled in Nyingchi, Tibet. The *A*. *thaliana* individuals were derived from two seeds of a single Col-0 plant. Three rice individuals, including one *O*. *sativa* L. cv. Pei-Ai 64s (PA1), two *O*. *sativa* ssp. indica cv. Kasalath (KA1), and cv. Dee-geo-woo-gen (DG1), were obtained from the International Rice Research Institute (IRRI). The plum trees were sampled in Nanjing. The shrub willow YAF1 was kindly provided by Jiangsu Forestry Science Academe in China. The woodland strawberry was obtained from Nanjing Agricultural University, which was the same accession (Hawaii 4) as the reference genome. The seeds of *B*. *distachyon* diploid inbred line Bd21 (WD2) were obtained from South China Agricultural University.

In total, 480 leaves were sampled from the terminal branches of 21 plants. For rice DG1, *B*. *distachyon* WD2, willow YAF1, plum MHG1, and peach PXL, 25, 8, 22, 32, and 13 root samples were collected, respectively. One bark sample was also prepared for PXL. Seven lemma samples were collected for WD2 before maturing. Four stem samples were obtained from strawberry FH1. For a wild peach tree GL2, 14 fruits were also sampled at the same date as its leaves. Those fruits were treated with gibberellin to accelerate germination. For a plum tree MHG1, 21 fruits were sampled 7 months after the leaf sampling.

The age of peach trees was estimated using a growth cone. The ages of wild peach trees were estimated based on work by Wang and colleagues [33].

DNA was extracted using the CTAB method [38]. About three-quarters of leaf DNA samples were extracted using a single leaf or part of a single leaf, weighing approximately 0.08–0.7 g. DNA samples for fruits of MHG1 were extracted from the seeds after carefully removing the seed coats. For progeny of GL2, the DNA samples were extracted from the leaves of seedlings after growing for approximately 1–2 months. The root and bark DNA samples were extracted after careful cleaning.

To obtain microscale plant samples, we used a Harris micropunch (0.5-mm diameter) to harvest a defined area of leaf. Genomic DNA of microscale samples was amplified with a Qiagen REPL-g single-cell kit following the kit instructions.

All plant DNA was fragmented into an insert size of about 300–350 bp and sequenced on the Illumina Hiseq4000 platform with 150-bp paired-end reads at BGI. Detailed statistics of sequencing results are provided in S1 Data.

### Sequencing and alignment

Whole-genome sequences and annotations for peach [39] and woodland strawberry [40] were downloaded from Genome Database for Rosaceae (GDR, https://www.rosaceae.org, version 2.0.a1). Both peach and woodland strawberry have a compact genome (about 240–260 M) and a qualified reference genome that is both of high accuracy and completeness. The peach genome was initially sequenced using Sanger reads and assembled into eight chromosomes [39], this being subsequently improved with additional linkage maps and NGS reads [41]. The woodland strawberry was initially sequenced with NGS reads, assembled into seven chromosomes [42], and improved by dense targeted capture linkage maps [43]. The reference genome and annotations for the plum tree [44] were downloaded from http://prunusmumegenome.bjfu.edu.cn, mirrored at https://github.com/lileiting/prunusmumegenome. The rice reference genome [45] was downloaded from the Rice Annotation Project Database (RAP-DB, http://rapdb.dna.affrc.go.jp/, version IRGSP-1.0), and the *Arabidopsis* reference genome [46] was obtained through The Arabidopsis Information Resource (TAIR, http://www.arabidopsis.org/, version 10).

Each sample was sequenced to a cleaned depth over 40× with qualified bases (base quality ≥ 20) over 90% after removing adaptors and low-quality reads (i.e., reads containing more than 50% low-quality bases). Cleaned reads were mapped to each reference genome using BWA-mem 0.7.10-r789 [47] with default settings. The resulting BAM files were then sorted and processed with MarkDuplicates in Picard package (version 1.114) to remove noninformative PCR duplicates. A local realignment step was also implemented using RealignerTargetCreator and IndelRealigner in GATK package version 3.5.0 [48] to reduce false variant calls due to alignment errors around insertions/deletions (indels).

The rice root samples were susceptible to bacterial contamination, which resulted in lower effective coverage. We excluded those samples with extremely low coverage (<45%) from further analysis and only used those samples to exclude false positives. The MHG1 individual was found to be from grafting; thus, the branches and the root were analyzed separately as independent systems.

### De novo mutation identification

Single-nucleotide variants (SNVs) and small indels were called using two distinct algorithms implemented in GATK: UnifiedGenotyper (UG) and HaplotypeCaller (HC). Only reads with a mapping quality over 20 (i.e., less than 1% error rate) were considered.

The initial candidate mutations were called by comparing the samples within the same branch against all other branches based on the branching topology (S1 Fig). A variant would be called as a candidate mutation if the allele is different from that in the comparator branches, which we presume reflects the ancestral state. This parallel comparison approach has been demonstrated to be robust against sequencing or mapping artifacts and has a relative low false-negative rate [26]. We compiled a series of criteria for filtering and evaluating the initial candidates (S1 Fig). Those criteria deal with all respects of sequencing, mapping, or calling errors.

First, we filtered candidates with low variant quality (quality score < 50 given in VCF file), low depth (no sample carries ≥5 putative mutated reads), or many missing calls (no variant calls in more than 5 samples). For mutations only found in a single sample, we required the focal sample (the sample assumed to carry a mutation) to contain at least five reads. For mutations shared by >1 sample, at least one sample should fit this criteria, and other samples should have no fewer than three reads carrying the same mutations. Variants that failed any of these criteria were assumed to be sequencing errors. We also removed candidates that were biased in read strands (only have forward or reverse strands), a signature of erroneous mapping artifacts from duplications. Second, we masked the remaining candidate sites that (1) have missing calls but no more than four samples, (2) have two or three reads with the same “mutated” alleles of base quality over 20 (termed as “mimic reads” hereafter) among all compared (control) samples, or (3) could only be captured by UG caller, this having the higher false-positive rate. Candidates passing all those criteria were considered the “confidence set,” otherwise they were treated as the “evaluation set” (S1 Fig).

We further manually investigated all candidates in the confidence set and part of the evaluation set, from most evidence to least. For each candidate mutation site, the Integrative Genomics Viewer (IGV) was applied to review the mapping states across all related samples. Loci found to have resulted from spurious mapping artifacts or contamination (detected by BLAST search in NCBI Nucleotide collection database using the aligned reads) were discarded. An additional round of inspection was performed for indels and SNVs around indels. We first extracted reads mapping to each candidate region and then realigned them to the reference sequence with ClustalW2. The regenerated alignments were saved in FASTA format and further revised in MEGA6 to get the best possible alignments. From this, we confirmed whether the candidate is a true variant or just a misalignment artifact. A candidate “mutation” is considered false if it (1) is an alignment artifact, mostly found in regions containing indels, regions divergent between the reference genome and analyzed genome, regions harboring large genomic rearrangements, duplications (which easily cause the wrong placement of reads), etc.; (2) is a preexisting variant (i.e., one actually present in all other samples) but happened to be called only in some samples (this situation is most likely due to some subtle differences in reads—e.g., slightly more sequencing errors in some—covering the candidate site between different samples, which cause some samples to pass the threshold and be called by the caller while others happened to fail); (3) is a contamination artifact, either from impurities on the sample’s surface or “sample bleeding (index hopping)” of multiplexed samples; or (4) resulted from sequencing errors, mainly found in regions with homopolymers or tandem repeats, which have dubious lengths among different samples and thus are less likely to be bona fide mutations.

It was found that the false discovery rate increased rapidly with increasing numbers of missed calls because there was more sequencing bias when relatively few samples were properly amplified and sequenced. The same situation was found for more “mimic reads.” Assuming all “mimic reads” were only from sequencing errors, for two mimic reads to be present in compared samples would require a probability less than (1% base error rate × 1/4 the same allele by chance)^2^ = 6.24 × 10^−6^. In practice, we found that the presence of more than one mimic read was mostly a signature of false mutation calls due to sequencing or mapping artifacts.

Another false-positive source was from misalignments around indels, which could be witnessed as a high error rate in candidates called by UG alone. The UG algorithm directly calls variants from the alignments and thus is capable of capturing most SNVs but could have a high false-positive call rate due to misalignment, especially around indel sites. The HC algorithm has fewer positive SNV calls and performs better in indel detection compared to UG, as HC implements a local reassembly algorithm. However, it was found that the HC caller occasionally lost a few SNVs, possibly because of the non-lossless GVCF mode or the reassembly process. We integrated results from the two callers in later analyses (S1 Fig). Through these mechanisms, we minimized both the false-positive rate and false-negative rate caused by the variant callers [49]. This was confirmed during the manual inspection stage. From the SNV mutations identified in this study, we found around 95.5% of SNVs could be called by both HC and UG callers, whereas 2.6% were only called by HC and 1.9% were only called by UG. For indel mutations, around 83.4% of indels were found in both call sets, whereas 15.8% could only be called by HC and 0.8% were only called by UG.

In general, the manual inspection suggested the confidence set could capture over 90% of candidate callable mutations (calculated as “Manually confirmed mutations in confidence set” / “Manually confirmed mutations in both confidence and evaluation set”) for base substitutions within accessible regions, whereas ignoring the evaluation set would only cause a false-negative rate of no more than 10%. As the evaluation set was manually investigated from higher confidence to lower, it was mostly likely that all callable mutations were captured in our analysis.

During the filtering stage, we also observed a certain number of cross-sample contaminations. Those contaminations were only found within different individuals that were sequenced in the same sequencing lane. A small number of reads that were believed to belong to one individual could be observed in another individual, especially in genomic regions with ultrahigh read coverage (e.g., >100×). This contamination was unlikely to be owing to early experimental mistakes, as each sample was processed independently during the DNA extraction and library construction stage. We could also rule out the possibility of read-assign errors, as the barcodes used for each sample were very different. Therefore, we conclude that those cross-contaminations are most likely a result of cluster-detection errors during the sequencing stage, known as “sample bleeding” [50]. These contaminants were removed by comparing against unrelated samples within the same lane.

The “topology-based” method could miss mutations that occurred originally in soma but were fixed across different branches. We searched for these heterogeneous sites that have a variant allele present in only some (“M”) of all “N” samples, hereafter referred as the frequency-based method (S1 and S8 Figs). The frequency-based method then compares every possible combination of M samples (focal samples supposed to carry the mutation allele) with the remaining N-M samples (treated as “control” samples) using the same criteria used in the “topology-based” method (like a comparison between two “branches”). Variant alleles present in over 0.8 * N samples were not considered as mutations because they (1) could well be preexisting variants for which not all samples were properly genotyped, owing to sequencing/mapping/calling biases, and (2) could not be distinguished from somatic recombination events (for further logic and illustration, see S8 Fig).

This method was more prone to various analytical errors, as the “control” samples were often inaccurate, which could miss true mutations if mutated samples were included in the control group (see Site4 in S8 Fig), while generating false candidates if insufficient samples were included in the control group (see Site5 in S8 Fig). Therefore, we only considered the most confident sites present in several samples defined as before but with no evidence in all other samples (e.g., no mimic reads allowed). Results from the frequency-based method could also be used to correct any errors in topology records (S1 Fig). For instance, the relationship of five primary branches of GL2 tree are almost indistinguishable (Fig 2A) and could only be treated as five independent branches, whereas, based on mutations shared between them, the frequency-based analysis suggested the branches B1 and B2 are actually ontogenetically closer (Fig 2B, same for B4 and B5).

Only substitutions and small indels (e.g., <100 bp) were investigated in this study. Substitutions include SNV and multiple-nucleotide variants (MNVs), whereas indels contain pure insertions (INSs), deletions (DELs), and complex replacements (RPLs; i.e., nonequal-size base substitutions). A full list of all identified mutations can be found in S2 Data.

Although new mutations are expected to be heterozygous, we did not filter with heterozygosity as a requirement; rather, we require a mutation to be different from the ancestral state. This decision was based on the premise that there exist several situations that could cause real mutations to be witnessed in an apparent or real “homozygous” state. These include subsequent somatic recombination leading to the loss of the nonmutated haplotype, sequencing bias in which only the mutated haplotype gets sequenced, mapping issues in which only the mutated haplotype is properly mapped, etc. As it happens, 99.0% of mutations we identified in this study were heterozygous.

### Estimation of false-negative/positive rates and callable sites

We used PCR and conventional Sanger sequencing to validate 89 mutations in 122 mutated samples, 59 progeny samples, and 274 control samples. The mutated samples were confirmed at a rate of 96.7% (118/122). The unconfirmed instances could reflect false positives or a failure of the PCR to amplify the mutant allele. No mutant allele was found in control samples.

The number of callable sites and the false-negative rate were estimated using a simulation method similar to that described previously [26,51]. The read-depth distribution for each group was based on the real mutations identified. For each tree, we generated 1,000 synthetic mutation sites in one or several branches according to each topology. The leaf and root samples were simulated separately because they had different read-depth distributions. The same pipelines were then used to detect these synthetic mutations. The fraction of callable sites in the genome for each tree was then estimated as the fraction of callable simulated mutated sites (S8 and S9 Tables).

### Calculation of the expected inheritance

Supposing a mutation is inherited by all the cells in a branch, and then each cell has a genotype of Aa (“A” is the wild allele, and “a” is the mutated allele). Fifty percent of all the gametes produced by this branch thus are expected to carry the “a” allele, and 50% carry “A.” The probability of the absence of “a” in all of the fertilized eggs would be (1/2)^*n*^, where *n* denotes the number of seeds.

### Statistical analysis

Statistics and correlation test were performed in R [52]. BM test was implemented in the R package “lawstat.”

## Supporting information

S1 Fig Flowchart of pipeline to identify somatic mutations. Take a tree with five primary branches (B1–B5) as an example, assuming a total of 40 leaves were sequenced (samples 1–8 were collected from B1, 9–16 were collected from B2, etc.) and two mutations were present in these leaves (Mut1 represents a mutation raised in B1 and fixed in nearly all samples collected from B1, and Mut2 represents a mutation raised before B1 and B5, which fixed in B5 but only presented in sample 2 of B1; samples carry the mutations were referred as “focal samples”). After obtaining the processed BAM files, the variants were called with GATK HC (in GVCF mode) to generate a multiple-sample VCF file. The variants in VCF file were refined with VarScan to obtain the accurate allele depths of SNVs and HC multiple calling mode to generate jointly adjusted indels. A candidate mutation was then called if the variant was unique to a single branch (branching topology—based approach), so Mut1 would be identified here. The candidate mutations were first filtered with “hard filtering” criteria and then ranked by evidences that could support its reliability (evidence collecting). To gain more evidences, another caller, UG, was added to reproduce the whole pipeline. Four basic evidences were applied to those mutation candidates, which categorized them into the “confidence set” if all evidences satisfied or “evaluation set” if any failed. For the confidence set, generally all candidates were manually assessed. For the evaluation set, the candidates were subsampled (candidates with more supporting evidences will be sampled in priority) for manually inspection. The filtering criteria and evidences would be further tuned if (1) the confidence set contains many candidates that fail manual inspection or (2) the evaluation set contain many candidates that could pass manual inspection. The topology-based approach could not identify Mut2, as it presents in multiple primary branches (B1 and B5). One way to detect Mut2 is to assign sample 3 and samples 31–40 into a group (a group with 11 samples or mutated sample frequency = 11) and compare this group to remaining 29 samples. As it is practically unknown which sample will carry Mut2, a workaround is to generate all combinations of samples into a focal group with supposed mutated frequency < 40 here and compare it with remaining samples (the frequency-based approach). This approach could reveal possible recording mistakes if sample 2 always shares mutations with samples from B5 but never with samples from B1. The branching topology would be corrected if such an error was found, and the whole pipeline would be rerun to obtain the finally confirmed mutation set. GVCF, genomic variant call format; HC, HaplotypeCaller; indel, insertion/deletion; SNV, single-nucleotide variant; UG, UnifiedGenotyper.
(TIF)

S2 Fig Schematic representation of different cuttings sampled from shrub willow (*S*. *suchowensis*). The shrub willow can grow leaf and root from cuttings; thus, a shoot—root group that share the exact same growth duration could be obtained. (A) Somatic mutations identified in leaf and root samples of each cutting. A total of eight shoot—root groups (cuts 1–8) were obtained from different cuttings of the individual YAF1. The somatic mutations identified for each group were given in S1 Table. (B) An example of the cutting (circled in part A) grown for a week. Each twig (approximately 20 cm long) was cut from the original tree and cultured in water. The leaf bud and root became visible in about 1 week. (C) Photo of the cutting during sampling stage. The leaf and root were collected for each twig (a shoot—root group) after it grew for about 1 month.
(TIF)

S3 Fig Distribution and inheritability of somatic mutations in branches of two *Arabidopsis* samples (A and B). The two *Arabidopsis* were sampled in a greenhouse at Nanjing University, Nanjing in China. Leaves from both rosette and stem (separated by dashed lines in the figure) were sampled. Seeds from shoot apex and axillary branch were also sampled to test whether a mutation could be found in its progeny (shown in callout). Only one mutation, numbered 6, was confirmed in the progeny of Col17 axillary branch 3–2.
(TIF)

S4 Fig Distribution and inheritability of somatic mutations in DG1, one of the three rice samples. Seeds from each branch were collected and grown into seedlings for PCR confirmation. Three somatic mutations (17 and 28–29; red) were confirmed in two seeds (e.g., S3 of 1-L1 in branch B1).
(TIF)

S5 Fig Distribution of somatic mutations in the other two rice samples (A) KA1 and (B) PA1. (TIF)

S6 Fig Distribution of somatic mutations in peach HY1. **This peach tree (*P*. *persica*) was sampled at Nanjing in China**. Two leaves (marked by dotted red circles) and two flowers (each with two petals sampled) from four different branches were analyzed in microscale after sampling by Harris micropunch (marked by white holes within leaf or petal in the figure, 500-μm diameter). The average cell size was estimated to be 6 × 18 μm = 108 μm^2^ averaged from about 10 cells measured under microscope. The peach leaf was supposed to consist of around nine layers of cells [53], from which one leaf micropunch sample was estimated to contain approximately 16,353 cells (500 μm^2^ × π / 4 / 108 μm^2^ × 9). However, given that only cells around the boundary could be lysed and used in DNA extraction, the effective cell number of each micropunch sample was estimated to be 785 (500 μm × π / 18 μm) to 2,356 (500 μm × π / 6 μm × 9). For the petal sample, the cell size and number is hard to measure because of its fragility; nonetheless, a rough estimation under microscope gives the similar size and number of layers compared to leaf. Only somatic mutations (substitutions) raised in each branch (i.e., mutations fixed in different leaves) were shown here; the within-leaf or within-petal differences were given in S5 Table.
(TIF)

S7 Fig Somatic substitution mutations identified in rice tissue culture samples. One panicle of rice (cultivar Nipponbare) individual “NIPB” (the P0 plant) was picked, and seeds from this panicle were induced into callus. The callus samples were further induced to generate subcultures (repeat once). After differentiation, the subcultures were grown into different lines (e.g., S8-8, S8-9, S2-9, etc.). Before differentiation, the weight of S8-b increased from 1.1 mg (1-mg subculture was estimated to contain approximately 106 cells through measuring cell numbers of callus sections) to 657.3 mg; assuming a constant density of subcultures, this gives a cell division number of 10 (= log_2_(657.3/1.1)). For the P0 plant, the leaves of different tillers were sampled for sequencing to identify somatic mutations raised during the growth of the plants. The regenerated plants were sequenced with at least one tiller (one leaf per tiller) to identify mutations raised during the culture process. The number within square brackets stand for identified point mutations. Mutations raised during culture process were marked as blue (numbers indicate mutations raised during each stage), whereas mutations raised in tiller samples were marked as red (numbers indicate accumulated mutations in each tiller). The S8-9 line has four regenerated plants sequenced, and the S8-10 line has two. For mutations shared among the regenerated plants of each line, they are most likely to be raised in the previous subculture process (marked by lightning). Therefore, a per-site and per-cell-division normalized substitution rate of 2.80 × 10^−8^ and 3.72 × 10^−8^ was estimated for the S8-9 and S8-10 lines, respectively, based on those shared mutations. Dashed lines stand for a few repeat steps not shown.
(TIF)

S8 Fig Comparison of topology- and frequency-based methods in calling somatic mutations. Assuming 10 samples (S1–S10) were collected from three primary branches (B1–B3) of a tree, each genomic sites could be summarized into five given situations (Site1–5). Site1 represents an invariant site where all 10 samples are of the same genotype. Site2 represents a variant site with extremely high allele frequency (M = 9) of “T.” It is possible that it is a true mutation in S1 (T→A) or even in S2–S10 (A→T) but is not distinguishable from technical artifacts as well as somatic recombination. Site3 represents a variant site with medium allele frequency (M < 8, A→T mutation in S1–S4). The frequency-based method first identifies these sites and then compares those mutated samples (S1–S4, marked by a red box) to other nonmutated controls (S5–S10, marked by a black box) to confirm whether it is reliable. This site could also be called by the topology-based method (compare B1 with B2 and B3). Site4 is the same as Site3, but add the technical artifacts into consideration. The “T” allele in S4 was only supported by two reads because of sequencing bias and thus could be genotyped as “A/A” by callers, whereas S6 happened to contain a sequencing error of “T.” This time, the frequency-based method would treat S1–S3 as mutated samples and S4–S10 as control samples and reject this site, as too many “T” alleles found in control samples would suggest this candidate is not reliable. The topology-based method could capture this because it could determine which “T” allele is more likely to be real. Site5 represents a variant site where S1, S4, S5, S7, S8, and S9 were genotyped with the same “T” allele (so these six samples will be considered as the “mutated” samples) but others are not (the four remaining samples will be considered as “control” samples). Only the frequency-based method could identify this site, as the mutated samples were from different primary branches. However, in practice, the confirmed results from the frequency-based method generally agreed well with those from the topology-based method. Sites like Site5 were found to be either (1) technical artifacts in which a few samples happened to be genotyped differently from others but were found to be identical in all samples when manually inspected (some samples in the control group, like S3 and S10, could have signatures for this; i.e., each with one identical read carries the same “T” allele but could be insufficient for a rejection when only limited control samples were available) (2) or putative recording errors in branching topologies.
(TIF)

S1 Table Somatic mutations identified in each root–shoot group of shrub willow. (DOCX)

S2 Table Mutations identified from nine meiotic progeny of peach GZ by whole-genome sequencing. DNA for TH-5 was directly extracted from seed after carefully removing the seed coat. Other seeds were germinated and grown into saplings for about 2–3 months until leaf sampling. A more detailed description was described by Xie and colleagues [26].
(DOCX)

S3 Table Mutations identified in the 21 meiotic progeny of *P*. *mume* MHG1 by whole-genome sequencing. DNA was directly extracted from the seed of each fruit after carefully removing the seed coats. Only the inherited mitotic mutations were detected because it is difficult to identify the de novo specific mutations for the outcrossed offspring between different parents of the plum trees. In addition, PCR and Sanger sequencing were used to identify the inherited mitotic mutations in 10 other samples (B1-1-1-1-FR1, B1-1-2-1-FR2, B1-2-1-FR1, B1-2-2-FR1, B2-3-FR1, B2-4-FR1, B2-5-FR2, B3-1-1-FR1, B3-1-2-FR1, and B3-1-3-FR1). Those fruits were germinated and growing into saplings. DNA was extracted from the leaf of saplings for each sample. A total of three inherited mutations (among nine mutation loci that could obtain valid results) were detected from 18 PCR and Sanger sequencings, and half of those progeny were found to inherit one or two somatic mutations.
(DOCX)

S4 Table Mutations identified in the meiotic progeny of *B*. *distachyon* WD2. In total, 16 progeny of WD2 were also whole-genome sequenced to identify the inherited somatic mutations and mutations putatively raised from the meiotic process. The inherited mutations identified here exactly match previous PCR verification results (Fig 3A). Assuming all specific mutations found in the progeny were raised in the meiotic process, the base mutation rate per generation per site of *B*. *distachyon* was estimated to be 3.34 × 10^−9^ (95% CI 2.24 × 10^−9^ to 4.80 × 10^−9^), and the indel mutation rate was about 6.92 × 10^−10^ (95% CI 2.54 × 10^−10^ to 1.51 × 10^−9^).
(DOCX)

S5 Table Sample-specific somatic mutations (substitutions) identified in peach HY1 micropunch samples. Two leaves and four flower petals from four branches were collected, and a total of 29 micropunch samples (each contain about 1,000 cells) were sequenced (26 samples were sequenced at about 40×; three leaf samples were sequenced to around 1,000×). Four petal samples were discarded because of very poor genome coverage (<10%). Petal samples displayed extremely high mutation numbers compared to leaf samples with similar sequencing coverage. Three leaf samples (L3-2, L3-6, L3-10) were sequenced to an ultrahigh depth of 1,000×. Three independent libraries were constructed for each sample to minimize putative bias in library preparation, and each library was sequenced to a raw depth of 400×. For each sample, one library was also sequenced at a moderate depth of 40×, similar to other samples. Mutations for the three samples were called using both the 40× sequencing data (numbers given in brackets) and all 1,000× data. For petal samples and 1,000× leaf samples, we used more-stringent criteria by requiring that (1) no identical mutation reads were found in other samples and (2) the mutation allele was not a preexisting polymorphism. The identified somatic mutations did increase as the sequencing depth increased (from 40× to 1,000×) in three leaf samples, but the total number was still very low, especially compared with petal samples.
(DOCX)

S6 Table Mutations identified in rice tissue culture samples. Ten leaves (T1–T4, T5–T11, one leaf per tiller) were sampled from the original parental plant. For regenerated plants, one or more leaves (one leaf per tiller) from arbitrary chosen tillers were sampled. The mutations were identified by comparing leaf between different tillers or different plants, as shown in S7 Fig.
(DOCX)

S7 Table Geographic location and sampling date of trees. (DOCX)

S8 Table Genome callable fractions of species with clear topologies. (DOCX)

S9 Table Genome callable fractions of species without clear topologies. (DOCX)

S1 Data Statistics of sequenced samples. (XLSX)

S2 Data List of identified mutation sites. (XLSX)

## Abbreviations

BM: Brunner-Munzel
RAM: root apical meristem
SAM: shoot apical meristem
