# Supplementary figures and images for: The architecture of intra-organism mutation rate variation in plants
Source: PLoS Biol. 2019 Apr 9;17(4):e3000191. doi: 10.1371/journal.pbio.3000191 (PMC6456163; doi:10.1371/journal.pbio.3000191)

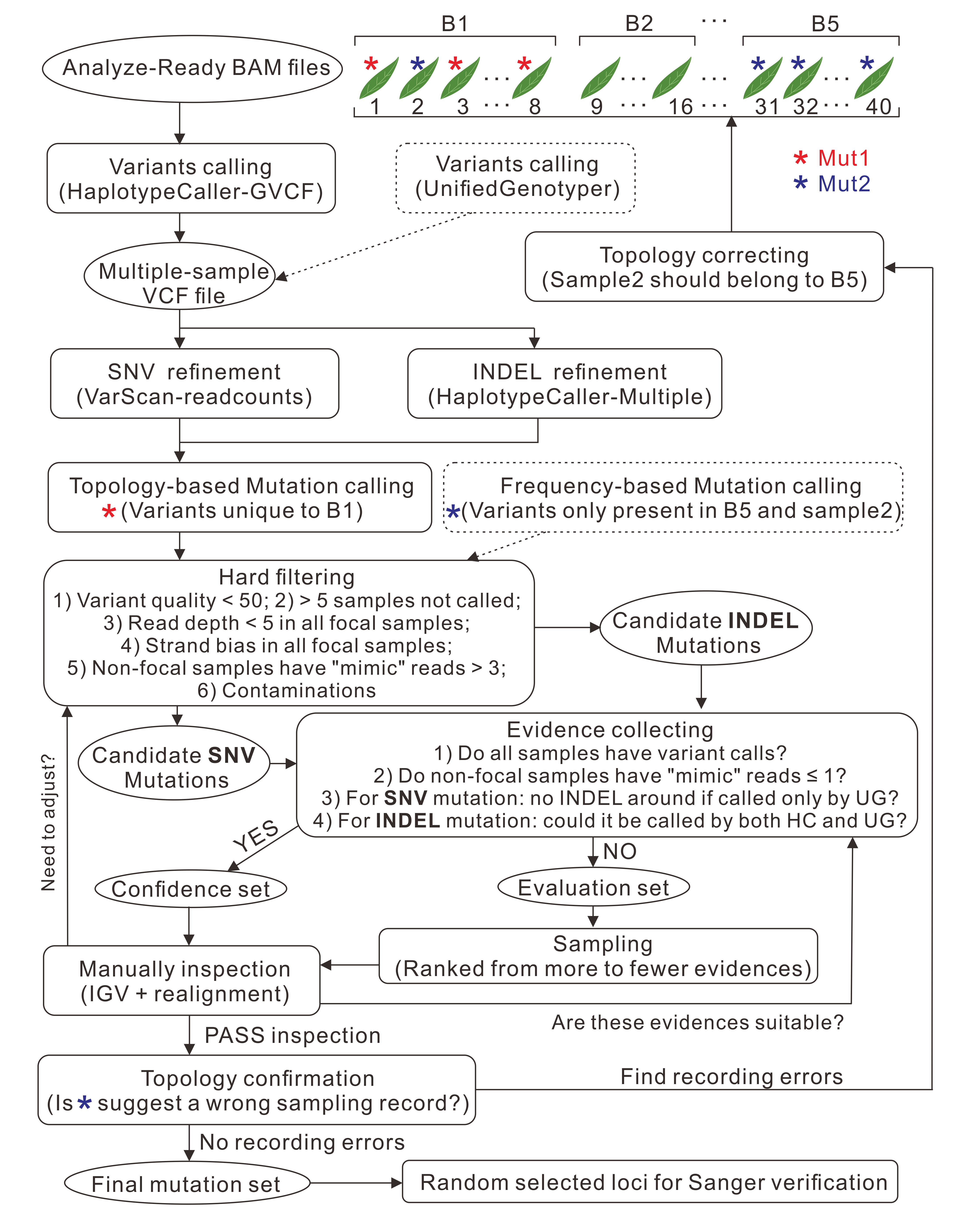

Supplement: S1 Fig — Take a tree with five primary branches (B1–B5) as an example, assuming a total of 40 leaves were sequenced (samples 1–8 were collected from B1, 9–16 were collected from B2, etc.) and two mutations were present in these leaves (Mut1 represents a mutation raised in B1 and fixed in nearly all samples collected from B1, and Mut2 represents a mutation raised before B1 and B5, which fixed in B5 but only presented in sample 2 of B1; samples carry the mutations were referred as “focal samples”). After obtaining the processed BAM files, the variants were called with GATK HC (in GVCF mode) to generate a multiple-sample VCF file. The variants in VCF file were refined with VarScan to obtain the accurate allele depths of SNVs and HC multiple calling mode to generate jointly adjusted indels. A candidate mutation was then called if the variant was unique to a single branch (branching topology—based approach), so Mut1 would be identified here. The candidate mutations were first filtered with “hard filtering” criteria and then ranked by evidences that could support its reliability (evidence collecting). To gain more evidences, another caller, UG, was added to reproduce the whole pipeline. Four basic evidences were applied to those mutation candidates, which categorized them into the “confidence set” if all evidences satisfied or “evaluation set” if any failed. For the confidence set, generally all candidates were manually assessed. For the evaluation set, the candidates were subsampled (candidates with more supporting evidences will be sampled in priority) for manually inspection. The filtering criteria and evidences would be further tuned if (1) the confidence set contains many candidates that fail manual inspection or (2) the evaluation set contain many candidates that could pass manual inspection. The topology-based approach could not identify Mut2, as it presents in multiple primary branches (B1 and B5). One way to detect Mut2 is to assign sample 3 and samples 31–40 into a group [file pbio.3000191.s001.tif]

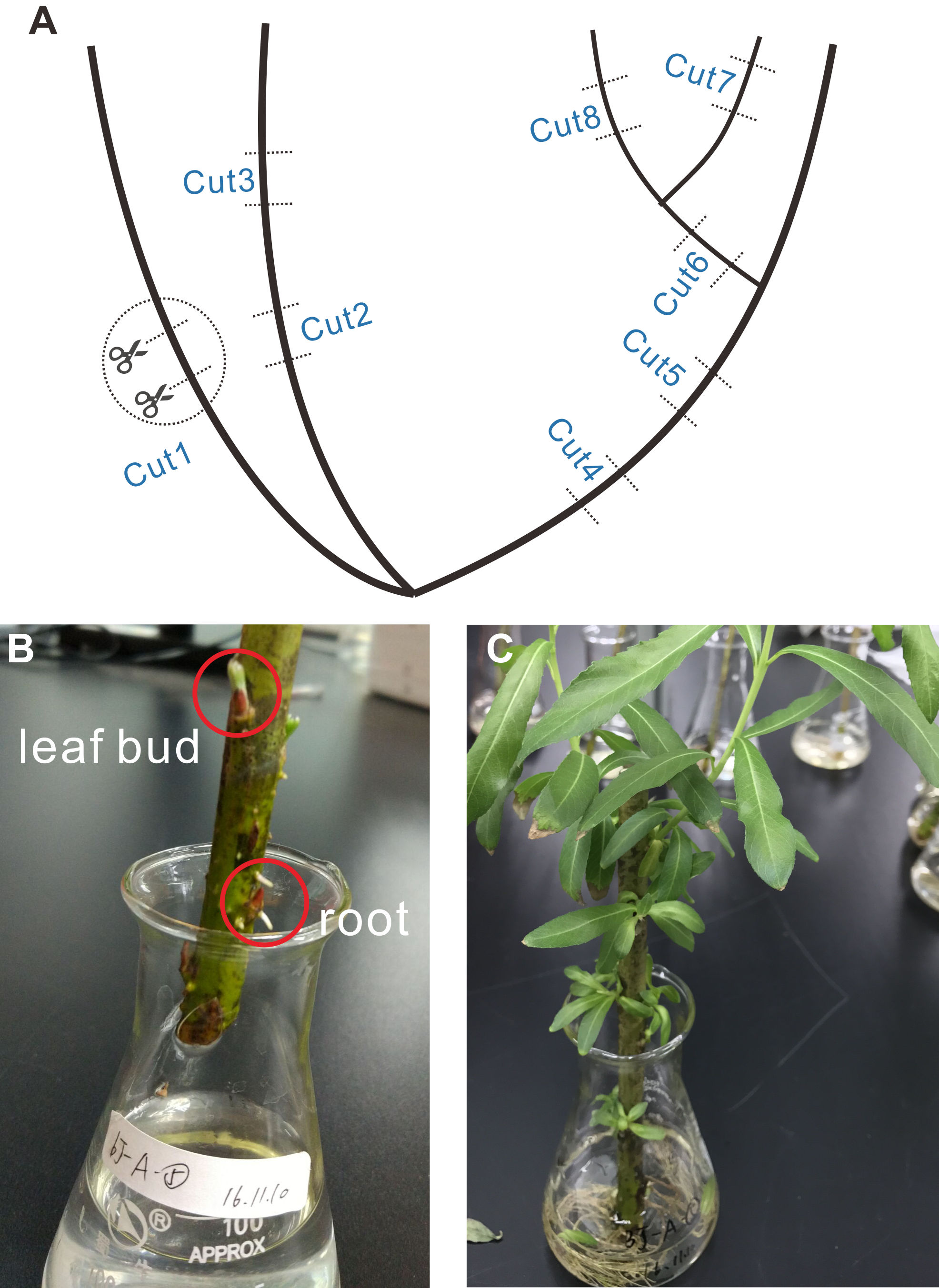

Supplement: S2 Fig — The shrub willow can grow leaf and root from cuttings; thus, a shoot—root group that share the exact same growth duration could be obtained. (A) Somatic mutations identified in leaf and root samples of each cutting. A total of eight shoot—root groups (cuts 1–8) were obtained from different cuttings of the individual YAF1. The somatic mutations identified for each group were given in S1 Table. (B) An example of the cutting (circled in part A) grown for a week. Each twig (approximately 20 cm long) was cut from the original tree and cultured in water. The leaf bud and root became visible in about 1 week. (C) Photo of the cutting during sampling stage. The leaf and root were collected for each twig (a shoot—root group) after it grew for about 1 month. (TIF) [file pbio.3000191.s002.tif]

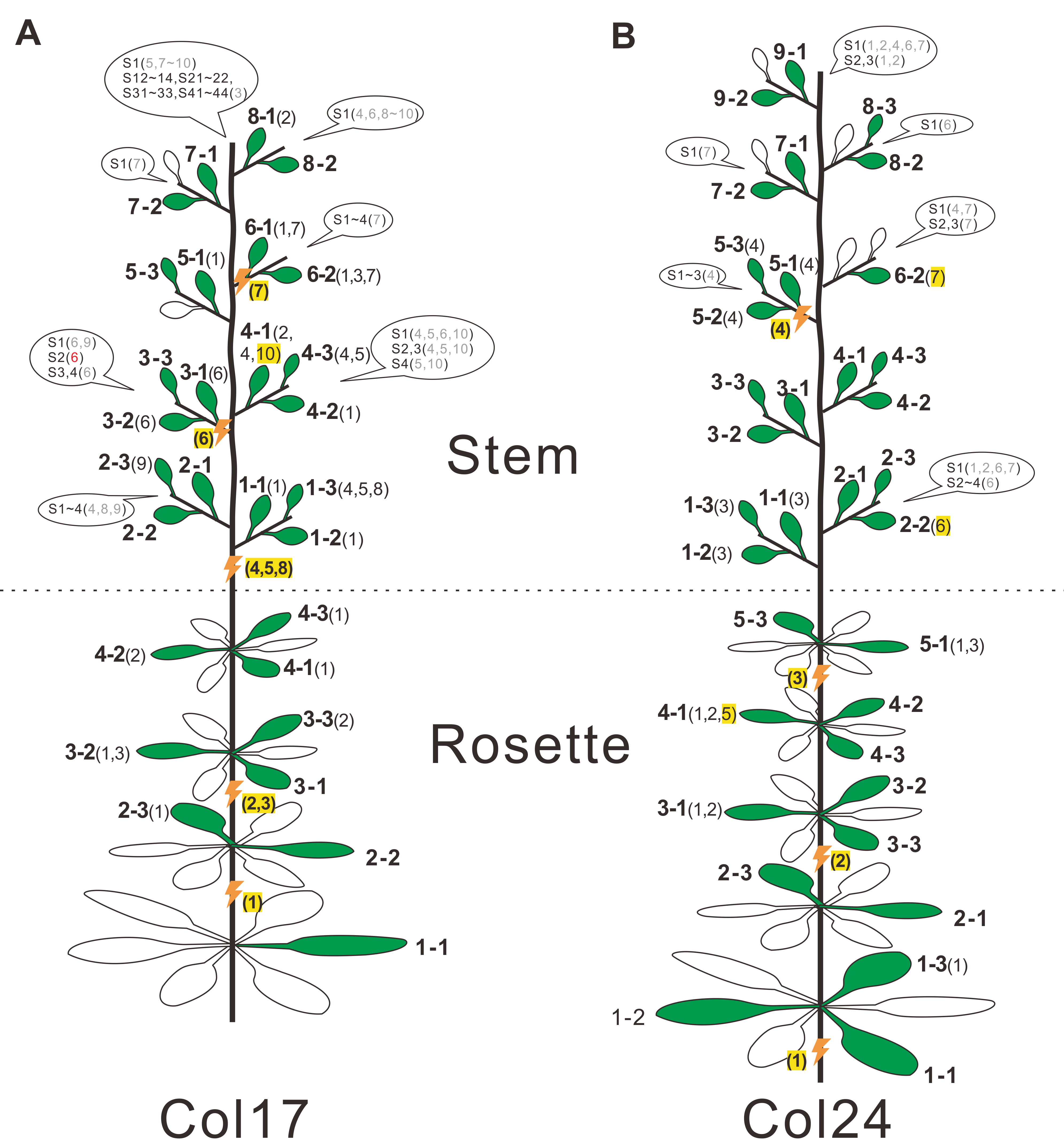

Supplement: S3 Fig — The two Arabidopsis were sampled in a greenhouse at Nanjing University, Nanjing in China. Leaves from both rosette and stem (separated by dashed lines in the figure) were sampled. Seeds from shoot apex and axillary branch were also sampled to test whether a mutation could be found in its progeny (shown in callout). Only one mutation, numbered 6, was confirmed in the progeny of Col17 axillary branch 3–2. (TIF) [file pbio.3000191.s003.tif]

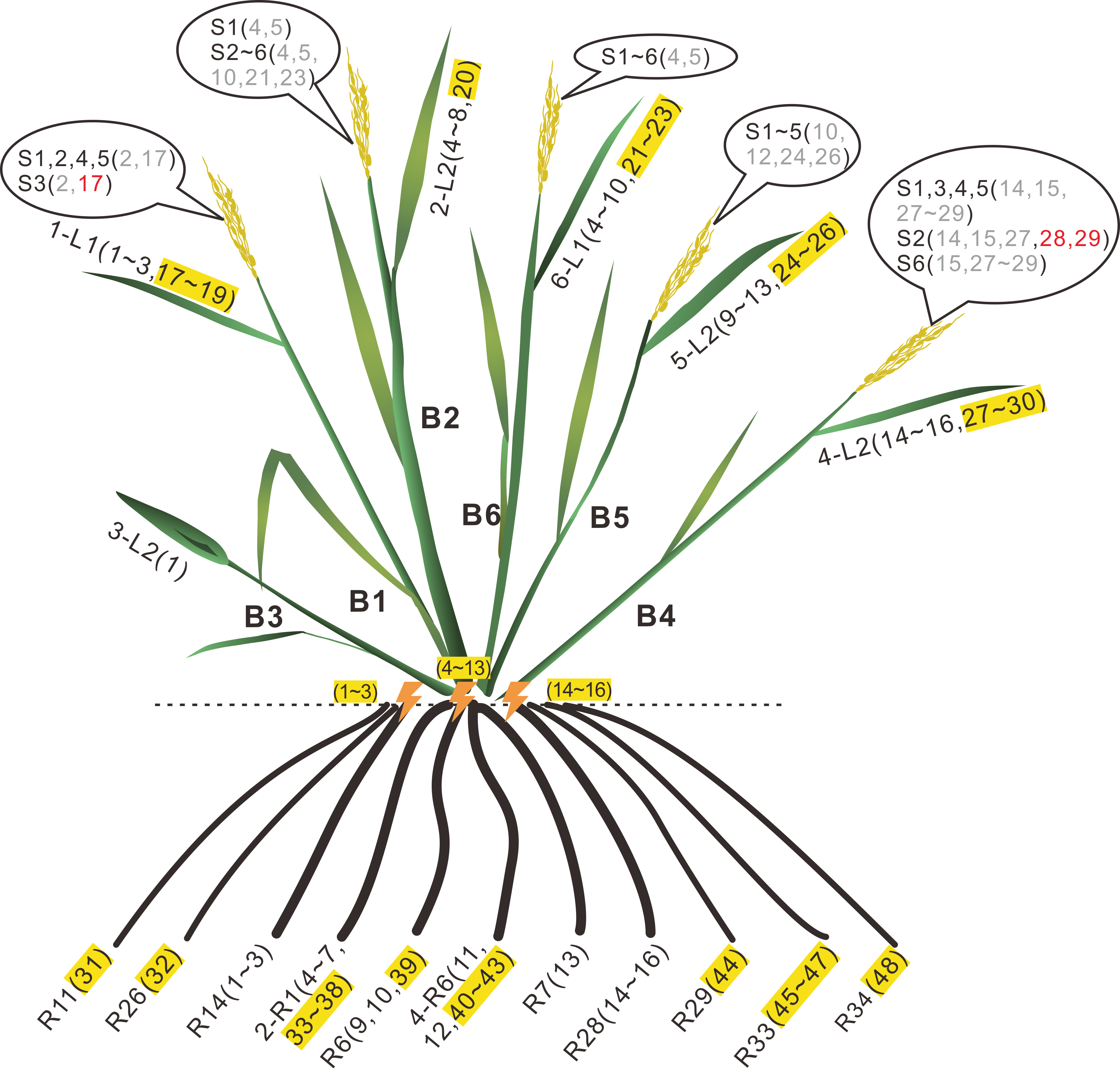

Supplement: S4 Fig — Seeds from each branch were collected and grown into seedlings for PCR confirmation. Three somatic mutations (17 and 28–29; red) were confirmed in two seeds (e.g., S3 of 1-L1 in branch B1). (TIF) [file pbio.3000191.s004.tif]

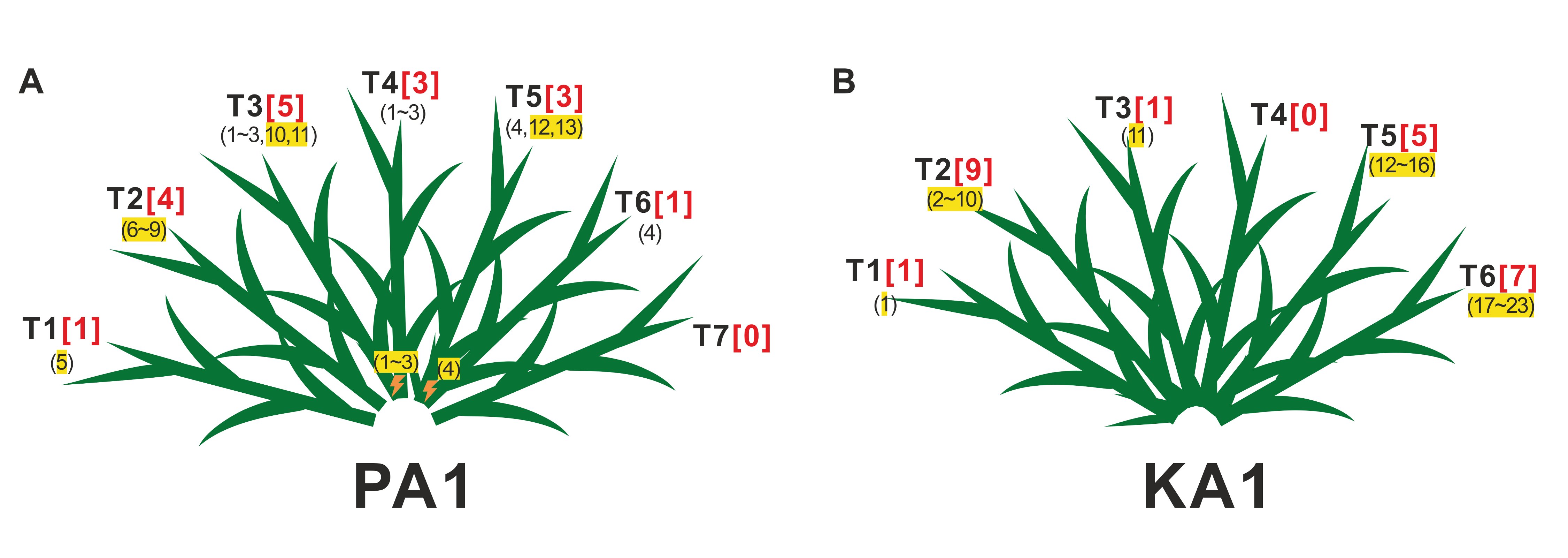

Supplement: S5 Fig — (TIF) [file pbio.3000191.s005.tif]

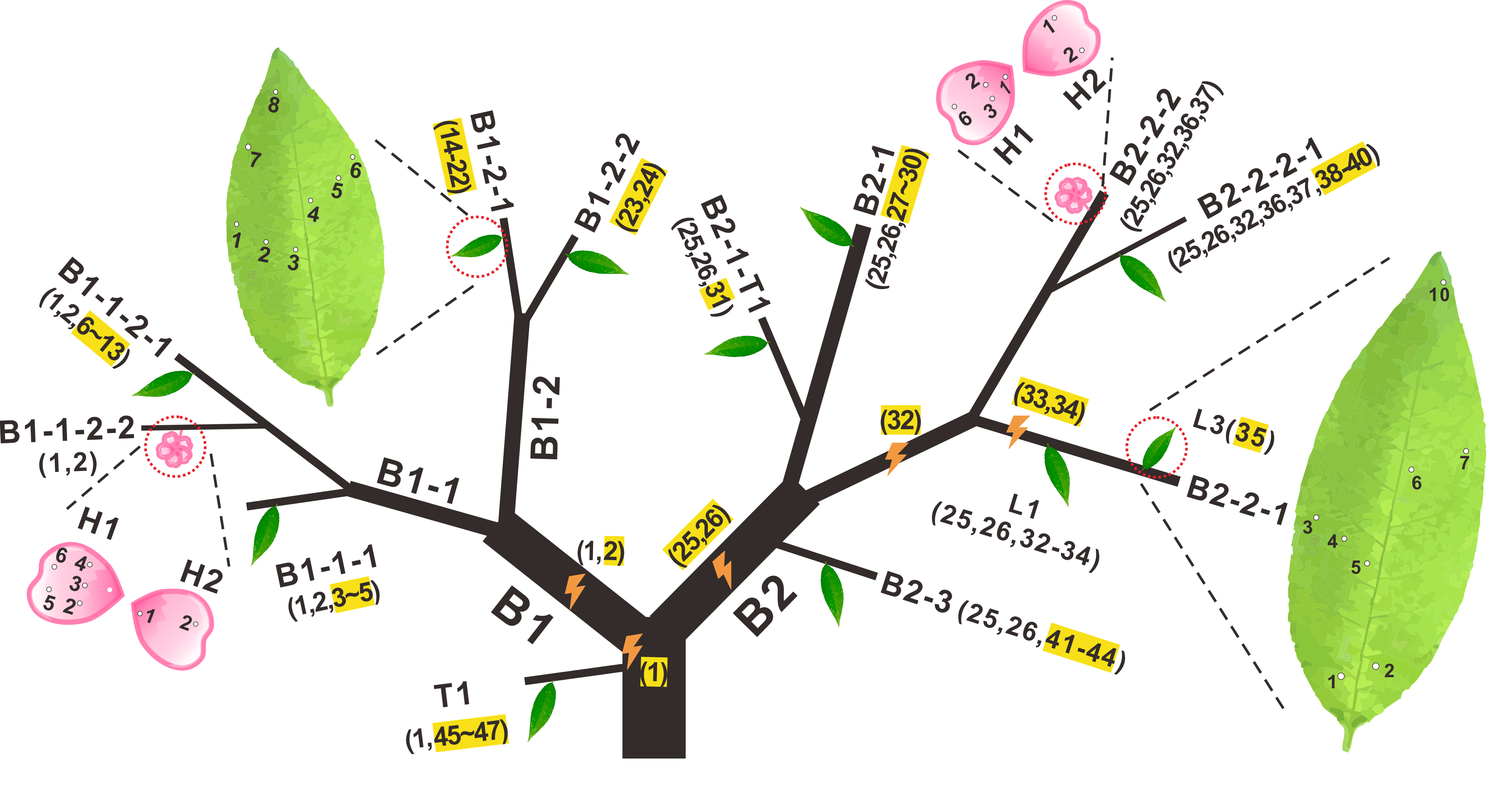

Supplement: S6 Fig — This peach tree (P. persica) was sampled at Nanjing in China. Two leaves (marked by dotted red circles) and two flowers (each with two petals sampled) from four different branches were analyzed in microscale after sampling by Harris micropunch (marked by white holes within leaf or petal in the figure, 500-μm diameter). The average cell size was estimated to be 6 × 18 μm = 108 μm2 averaged from about 10 cells measured under microscope. The peach leaf was supposed to consist of around nine layers of cells [53], from which one leaf micropunch sample was estimated to contain approximately 16,353 cells (500 μm2 × π / 4 / 108 μm2 × 9). However, given that only cells around the boundary could be lysed and used in DNA extraction, the effective cell number of each micropunch sample was estimated to be 785 (500 μm × π / 18 μm) to 2,356 (500 μm × π / 6 μm × 9). For the petal sample, the cell size and number is hard to measure because of its fragility; nonetheless, a rough estimation under microscope gives the similar size and number of layers compared to leaf. Only somatic mutations (substitutions) raised in each branch (i.e., mutations fixed in different leaves) were shown here; the within-leaf or within-petal differences were given in S5 Table. (TIF) [file pbio.3000191.s006.tif]

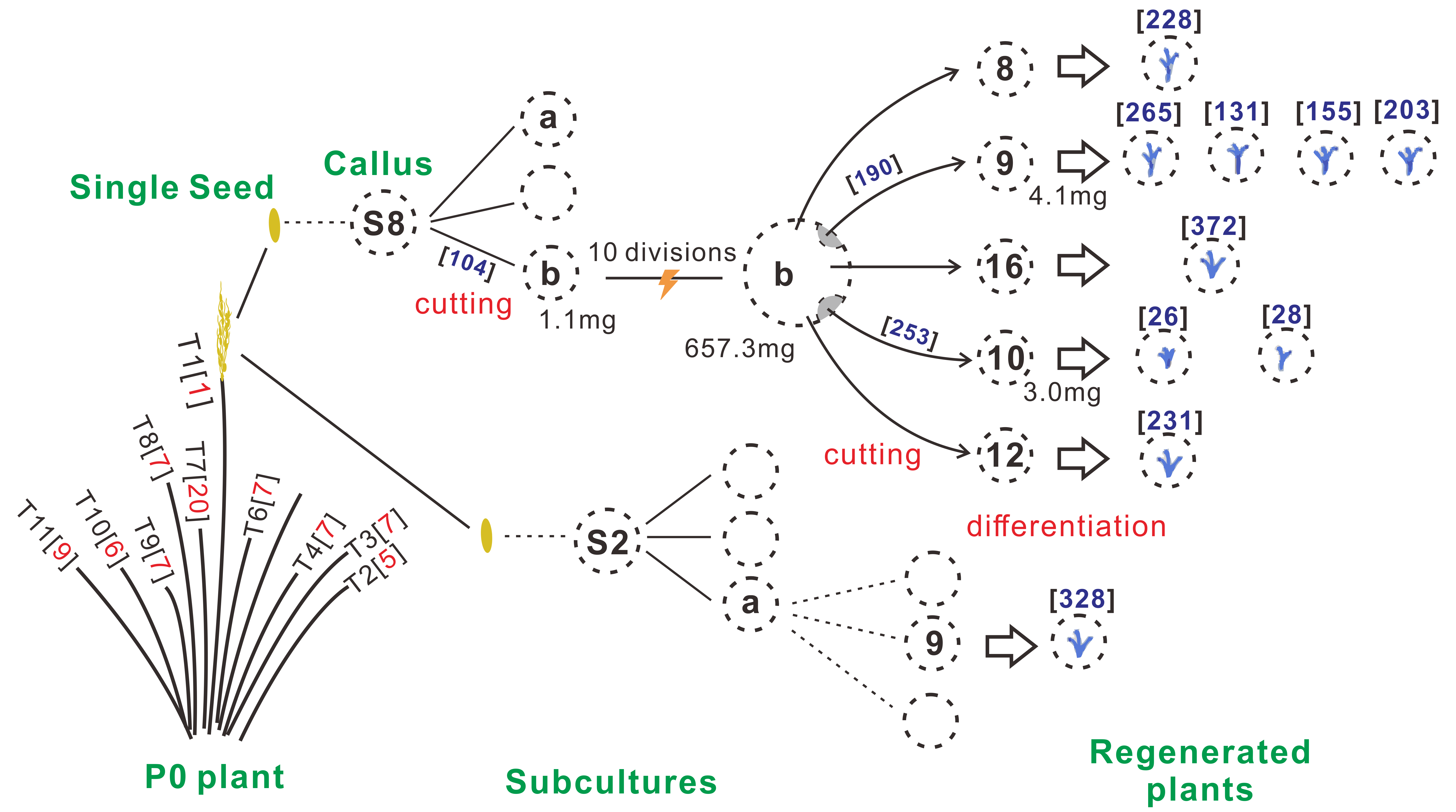

Supplement: S7 Fig — One panicle of rice (cultivar Nipponbare) individual “NIPB” (the P0 plant) was picked, and seeds from this panicle were induced into callus. The callus samples were further induced to generate subcultures (repeat once). After differentiation, the subcultures were grown into different lines (e.g., S8-8, S8-9, S2-9, etc.). Before differentiation, the weight of S8-b increased from 1.1 mg (1-mg subculture was estimated to contain approximately 106 cells through measuring cell numbers of callus sections) to 657.3 mg; assuming a constant density of subcultures, this gives a cell division number of 10 (= log2(657.3/1.1)). For the P0 plant, the leaves of different tillers were sampled for sequencing to identify somatic mutations raised during the growth of the plants. The regenerated plants were sequenced with at least one tiller (one leaf per tiller) to identify mutations raised during the culture process. The number within square brackets stand for identified point mutations. Mutations raised during culture process were marked as blue (numbers indicate mutations raised during each stage), whereas mutations raised in tiller samples were marked as red (numbers indicate accumulated mutations in each tiller). The S8-9 line has four regenerated plants sequenced, and the S8-10 line has two. For mutations shared among the regenerated plants of each line, they are most likely to be raised in the previous subculture process (marked by lightning). Therefore, a per-site and per-cell-division normalized substitution rate of 2.80 × 10^−8^ and 3.72 × 10^−8^ was estimated for the S8-9 and S8-10 lines, respectively, based on those shared mutations. Dashed lines stand for a few repeat steps not shown. (TIF) [file pbio.3000191.s007.tif]

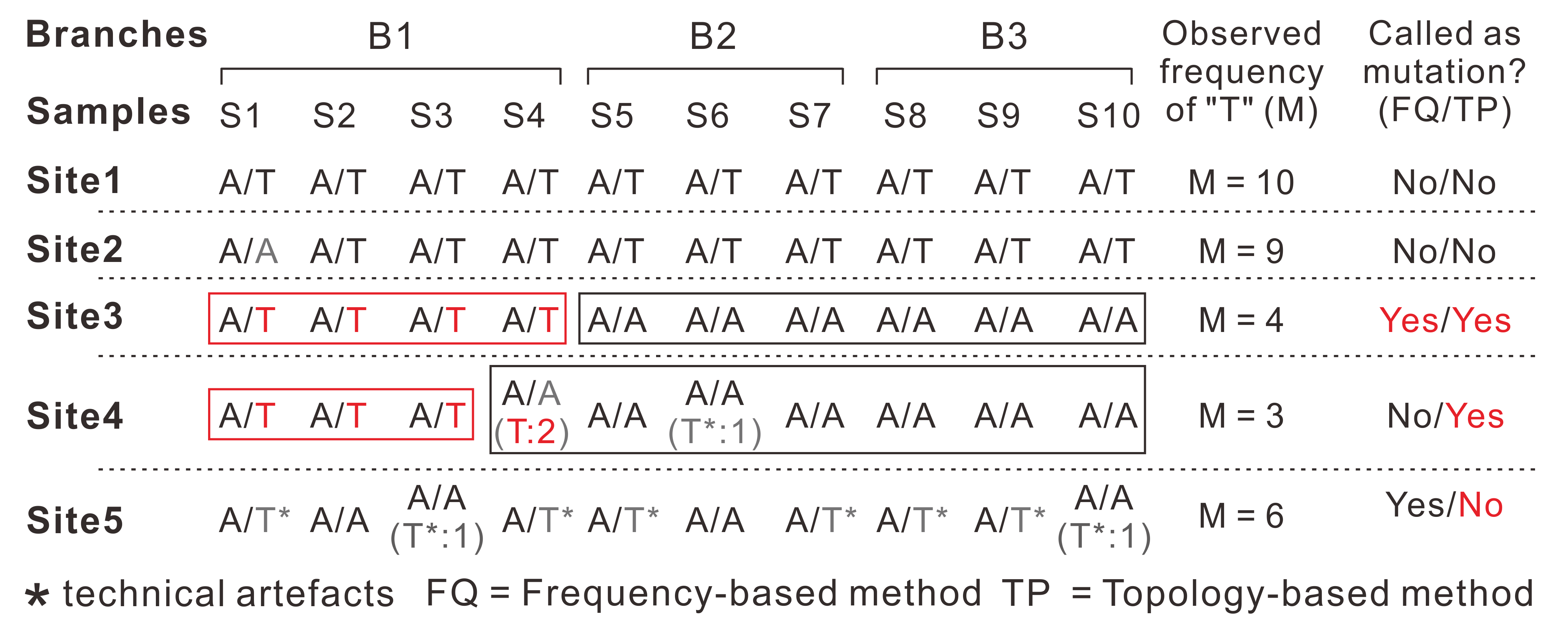

Supplement: S8 Fig — Assuming 10 samples (S1–S10) were collected from three primary branches (B1–B3) of a tree, each genomic sites could be summarized into five given situations (Site1–5). Site1 represents an invariant site where all 10 samples are of the same genotype. Site2 represents a variant site with extremely high allele frequency (M = 9) of “T.” It is possible that it is a true mutation in S1 (T→A) or even in S2–S10 (A→T) but is not distinguishable from technical artifacts as well as somatic recombination. Site3 represents a variant site with medium allele frequency (M < 8, A→T mutation in S1–S4). The frequency-based method first identifies these sites and then compares those mutated samples (S1–S4, marked by a red box) to other nonmutated controls (S5–S10, marked by a black box) to confirm whether it is reliable. This site could also be called by the topology-based method (compare B1 with B2 and B3). Site4 is the same as Site3, but add the technical artifacts into consideration. The “T” allele in S4 was only supported by two reads because of sequencing bias and thus could be genotyped as “A/A” by callers, whereas S6 happened to contain a sequencing error of “T.” This time, the frequency-based method would treat S1–S3 as mutated samples and S4–S10 as control samples and reject this site, as too many “T” alleles found in control samples would suggest this candidate is not reliable. The topology-based method could capture this because it could determine which “T” allele is more likely to be real. Site5 represents a variant site where S1, S4, S5, S7, S8, and S9 were genotyped with the same “T” allele (so these six samples will be considered as the “mutated” samples) but others are not (the four remaining samples will be considered as “control” samples). Only the frequency-based method could identify this site, as the mutated samples were from different primary branches. However, in practice, the confirmed results from the frequency-based method generally agreed well with those from [file pbio.3000191.s008.tif]
