# Supplementary material for: The architecture of intra-organism mutation rate variation in plants
Source: PLoS Biol. 2019 Apr 9;17(4):e3000191. doi: 10.1371/journal.pbio.3000191 (PMC6456163; doi:10.1371/journal.pbio.3000191)
Supplement: S1 Table — (DOCX) [file pbio.3000191.s009.docx]

| **Cuttings** | **Leaf ID** | **Substitutions** | **Root ID** | **Substitutions** |
| --- | --- | --- | --- | --- |
| Cut1 | 5_L1 | 0 | 5-R1 | 1 |
|  | 5_L2 | 0 | 5-R2 | 3 |
|  | 5_L3 | 1 | 5-R3 | 0 |
| Cut2 | 24_L1 | 1 | 24-R1 | 1 |
|  | 24_L2 | 2 | 24-R2 | 0 |
|  | 24_L3 | 2 | 24-R3 | 6 |
| Cut3 | 25_L1 | 2 | 25-R1 | 1 |
|  | 25_L2 | 1 | 25-R2 | 2 |
|  | 25_L3 | 2 | 25-R3 | 5 |
| Cut4 | 34_L1 | 3 | 34-R1 | 2 |
|  | 34_L3 | 2 | 34-R2 | 0 |
|  | 34_L4 | 0 | 34-R3 | 2 |
| Cut5 | 35_L1 | 0 | 35-R1 | 1 |
|  | 35_L2 | 1 | 35-R2 | 5 |
|  | 35_L3 | 0 | 35-R3 | 0 |
| Cut6 | 47_L3 | 1 | 47_R1 | 2 |
|  |  |  | 47_R2 | 7 |
| Cut7 | 51_L1 | 0 | 51_R3 | 3 |
|  | 51_L2 | 0 | 51_R4 | 3 |
| Cut8 | 55_L1 | 2 | 55_R1 | 3 |
|  |  |  | 55_R2 | 0 |
| Mean (95% C.I.) |  | 1.05 (0.64-1.63) |  | 2.24 (1.64-2.98) |
| Per year per site rate (×10^-9^) | | 2.16 (1.32-3.33) |  | 5.17 (3.80-6.87) |
