# Supplementary material for: The architecture of intra-organism mutation rate variation in plants
Source: PLoS Biol. 2019 Apr 9;17(4):e3000191. doi: 10.1371/journal.pbio.3000191 (PMC6456163; doi:10.1371/journal.pbio.3000191)
Supplement: S2 Table — DNA for TH-5 was directly extracted from seed after carefully removing the seed coat. Other seeds were germinated and grown into saplings for about 2–3 months until leaf sampling. A more detailed description was described by Xie and colleagues [26]. (DOCX) [file pbio.3000191.s010.docx]

| **Samples** | **Inherited mutations** | | | ***De novo* mutations** | | | **Proportion of the inherited mutations** |
| --- | --- | --- | --- | --- | --- | --- | --- |
|  | **Substitutions** | **Indels** | **All** | **Substitutions** | **Indels** | **All** |  |
| TH-5 | 6 | 1 | 7 | 4 | 4 | 8 | 7/15(47%) |
| TH-S1 | 6 | 1 | 7 | 2 | 1 | 3 | 7/10(70%) |
| TH-S2 | 6 | 2 | 8 | 1 | 0 | 1 | 8/9(89%) |
| TH-S3 | 5 | 1 | 6 | 4 | 0 | 4 | 6/10(60%) |
| TH-S4 | 3 | 1 | 4 | 5 | 0 | 5 | 4/9(44%) |
| TH-S5 | 5 | 0 | 5 | 4 | 1 | 5 | 5/10(50%) |
| TH-S7 | 5 | 2 | 7 | 1 | 0 | 1 | 7/8(88%) |
| TH-S8 | 8 | 1 | 9 | 2 | 1 | 3 | 9/12(75%) |
| TH-S9 | 7 | 1 | 8 | 2 | 0 | 2 | 8/10(80%) |
| **Mean** | 5.67 | 1.11 | 6.78 | 2.78 | 0.78 | 3.56 | 61/93(66%) |
