# Supplementary material for: The architecture of intra-organism mutation rate variation in plants
Source: PLoS Biol. 2019 Apr 9;17(4):e3000191. doi: 10.1371/journal.pbio.3000191 (PMC6456163; doi:10.1371/journal.pbio.3000191)
Supplement: S3 Table — DNA was directly extracted from the seed of each fruit after carefully removing the seed coats. Only the inherited mitotic mutations were detected because it is difficult to identify the de novo specific mutations for the outcrossed offspring between different parents of the plum trees. In addition, PCR and Sanger sequencing were used to identify the inherited mitotic mutations in 10 other samples (B1-1-1-1-FR1, B1-1-2-1-FR2, B1-2-1-FR1, B1-2-2-FR1, B2-3-FR1, B2-4-FR1, B2-5-FR2, B3-1-1-FR1, B3-1-2-FR1, and B3-1-3-FR1). Those fruits were germinated and growing into saplings. DNA was extracted from the leaf of saplings for each sample. A total of three inherited mutations (among nine mutation loci that could obtain valid results) were detected from 18 PCR and Sanger sequencings, and half of those progeny were found to inherit one or two somatic mutations. (DOCX) [file pbio.3000191.s011.docx]

| **Seed ID** | **Mitotic mutations in the branch of the fruit (progeny)** | | | **Inherited mitotic mutations in the progeny** | | |
| --- | --- | --- | --- | --- | --- | --- |
|  | **Substitutions** | **Indels** | **All** | **Substitutions** | **Indels** | **All** |
| B1-1-1-1-2S | 10 | 3 | 13 | 6 | 1 | 7 |
| B1-1-2-1-2-1S | 11 | 3 | 14 | 3 | 0 | 3 |
| B1-1-2-2-2S | 11 | 3 | 14 | 7 | 1 | 8 |
| B1-2-1-4S | 13 | 2 | 15 | 5 | 0 | 5 |
| B1-2-2-1-1S | 10 | 0 | 10 | 4 | 0 | 4 |
| B1-2-2-1-2S | 10 | 0 | 10 | 3 | 0 | 3 |
| B1-2-2-2-2S | 10 | 0 | 10 | 3 | 0 | 3 |
| B1-2-2-2-3-1S | 10 | 0 | 10 | 4 | 0 | 4 |
| B1-2-2-3-1S | 10 | 0 | 10 | 3 | 0 | 3 |
| B1-2-2-3-2S | 10 | 0 | 10 | 2 | 0 | 2 |
| B1-2-2-3-3-1S | 10 | 0 | 10 | 4 | 0 | 4 |
| B2-2-T1-2S | 7 | 4 | 11 | 1 | 0 | 1 |
| B2-4-1-1S | 11 | 7 | 18 | 3 | 2 | 5 |
| B2-4-2S | 12 | 7 | 19 | 3 | 0 | 3 |
| B2-5-1-1S | 8 | 10 | 18 | 2 | 4 | 6 |
| B2-5-1-2S | 8 | 10 | 18 | 3 | 2 | 5 |
| B2-5-2-1S | 8 | 10 | 18 | 2 | 2 | 4 |
| B2-5-2-2S | 8 | 10 | 18 | 3 | 0 | 3 |
| B3-2-1-2-2S | 4 | 2 | 6 | 0 | 1 | 1 |
| B3-2-2-1S | 1 | 2 | 3 | 1 | 0 | 1 |
| B3-2-2-4S | 1 | 2 | 3 | 1 | 0 | 1 |
| **Mean** | 8.71 | 3.57 | 12.29 | 3.00 | 0.62 | 3.62 |
