# Supplementary material for: The architecture of intra-organism mutation rate variation in plants
Source: PLoS Biol. 2019 Apr 9;17(4):e3000191. doi: 10.1371/journal.pbio.3000191 (PMC6456163; doi:10.1371/journal.pbio.3000191)
Supplement: S4 Table — In total, 16 progeny of WD2 were also whole-genome sequenced to identify the inherited somatic mutations and mutations putatively raised from the meiotic process. The inherited mutations identified here exactly match previous PCR verification results (Fig 3A). Assuming all specific mutations found in the progeny were raised in the meiotic process, the base mutation rate per generation per site of B. distachyon was estimated to be 3.34 × 10^−9^ (95% CI 2.24 × 10^−9^ to 4.80 × 10^−9^), and the indel mutation rate was about 6.92 × 10^−10^ (95% CI 2.54 × 10^−10^ to 1.51 × 10^−9^). (DOCX) [file pbio.3000191.s012.docx]

| **Seed ID** | **Inherited somatic mutations** | **Specific mutations in the progeny** | **Proportion of the inherited mutations** |
| --- | --- | --- | --- |
| B1-1-P3-S6 | 0 | 2 | 0% |
| B2-1-1-P2-S1 | 0 | 0 | NA |
| B2-1-2-1-P2-S2 | 0 | 4 | 0% |
| B2-1-2-1-P2-S3 | 0 | 3 | 0% |
| B2-1-2-1-P3-S4 | 0 | 4 | 0% |
| B2-1-2-1-P3-S5 | 0 | 2 | 0% |
| B2-1-2-2-P3-S1 | 3 | 4 | 43% |
| B2-1-2-2-P3-S3 | 2 | 1 | 67% |
| B2-1-2-2-P3-S4 | 1 | 2 | 33% |
| B2-1-2-2-P3-S5 | 2 | 3 | 40% |
| B2-2-P2-S5 | 1 | 1 | 50% |
| B3-1-P-S3 | 0 | 2 | 0% |
| B3-2-P2-S1 | 0 | 0 | NA |
| B3-2-P2-S3 | 2 | 2 | 50% |
| B3-2-P3-S1 | 0 | 2 | 0% |
| B3-3-P-S4 | 0 | 3 | 0% |
| **Mean** | 0.69 | 2.19 | 24% |
