# Supplementary material for: The architecture of intra-organism mutation rate variation in plants
Source: PLoS Biol. 2019 Apr 9;17(4):e3000191. doi: 10.1371/journal.pbio.3000191 (PMC6456163; doi:10.1371/journal.pbio.3000191)
Supplement: S5 Table — Two leaves and four flower petals from four branches were collected, and a total of 29 micropunch samples (each contain about 1,000 cells) were sequenced (26 samples were sequenced at about 40×; three leaf samples were sequenced to around 1,000×). Four petal samples were discarded because of very poor genome coverage (<10%). Petal samples displayed extremely high mutation numbers compared to leaf samples with similar sequencing coverage. Three leaf samples (L3-2, L3-6, L3-10) were sequenced to an ultrahigh depth of 1,000×. Three independent libraries were constructed for each sample to minimize putative bias in library preparation, and each library was sequenced to a raw depth of 400×. For each sample, one library was also sequenced at a moderate depth of 40×, similar to other samples. Mutations for the three samples were called using both the 40× sequencing data (numbers given in brackets) and all 1,000× data. For petal samples and 1,000× leaf samples, we used more-stringent criteria by requiring that (1) no identical mutation reads were found in other samples and (2) the mutation allele was not a preexisting polymorphism. The identified somatic mutations did increase as the sequencing depth increased (from 40× to 1,000×) in three leaf samples, but the total number was still very low, especially compared with petal samples. (DOCX) [file pbio.3000191.s013.docx]

| **Tissue** | **Branch** | **Petal / Leaf** | **Sample** | **Substitutions (Reads ≥ 5)** | **Substitutions (Reads ≥ 10)** |
| --- | --- | --- | --- | --- | --- |
| Flower | B1-1-2-2 | H1 | H1-2 | 779 | 129 |
|  |  |  | H1-4 | 6149 | 2744 |
|  |  | H2 | H2-1 | 667 | 163 |
|  |  |  | H2-2 | 1337 | 380 |
|  | B2-2-2 | H1 | H1-1 | 185 | 39 |
|  |  |  | H1-2 | 244 | 51 |
|  |  |  | H1-3 | 88 | 5 |
|  |  |  | H1-6 | 389 | 82 |
|  |  | H2 | H2-2 | 4267 | 2010 |
| Leaf | B1-2-1-2 | L1 | L1-1 | 2 | 0 |
|  |  |  | L1-2 | 3 | 0 |
|  |  |  | L1-3 | 4 | 0 |
|  |  |  | L1-4 | 2 | 1 |
|  |  |  | L1-5 | 0 | 0 |
|  |  |  | L1-6 | 22 | 0 |
|  |  |  | L1-7 | 9 | 1 |
|  |  |  | L1-8 | 1 | 0 |
|  | B2-2-1 | L3 | L3-1 | 0 | 0 |
|  |  |  | L3-3 | 1 | 0 |
|  |  |  | L3-4 | 1 | 0 |
|  |  |  | L3-5 | 0 | 0 |
|  |  |  | L3-7 | 2 | 1 |
|  |  | L3 (1000×) | L3-2 | 5 (0) | 2 (0) |
|  |  |  | L3-6 | 5 (1) | 1 (1) |
|  |  |  | L3-10 | 2 (0) | 0 (0) |
