# Supplementary material for: The architecture of intra-organism mutation rate variation in plants
Source: PLoS Biol. 2019 Apr 9;17(4):e3000191. doi: 10.1371/journal.pbio.3000191 (PMC6456163; doi:10.1371/journal.pbio.3000191)
Supplement: S6 Table — Ten leaves (T1–T4, T5–T11, one leaf per tiller) were sampled from the original parental plant. For regenerated plants, one or more leaves (one leaf per tiller) from arbitrary chosen tillers were sampled. The mutations were identified by comparing leaf between different tillers or different plants, as shown in S7 Fig. (DOCX) [file pbio.3000191.s014.docx]

| **Stage** | **Samples** | **Accumulated Substitutions** |
| --- | --- | --- |
| Mutations raised in parental tillers | T1 | 1 |
|  | T2 | 5 |
|  | T3 | 7 |
|  | T4 | 7 |
|  | T6 | 7 |
|  | T7 | 20 |
|  | T8 | 7 |
|  | T9 | 7 |
|  | T10 | 6 |
|  | T11 | 9 |
|  | Mean (95% C.I.) | 7.60 (5.99-9.51) |
| Mutations raised during tissue culturing | S2-9-2 | 328 |
|  | S8-10-3 | 380 |
|  | S8-10-4 | 380 |
|  | S8-12 | 262 |
|  | S8-16-1 | 374 |
|  | S8-8-1 | 239 |
|  | S8-9-2 | 276 |
|  | S8-9-3 | 419 |
|  | S8-9-5 | 443 |
|  | S8-9-6 | 469 |
|  | Mean (95% C.I.) | 357.0 (345.5-368.9) |
