# Supplementary material for: The architecture of intra-organism mutation rate variation in plants
Source: PLoS Biol. 2019 Apr 9;17(4):e3000191. doi: 10.1371/journal.pbio.3000191 (PMC6456163; doi:10.1371/journal.pbio.3000191)
Supplement: S7 Table — (DOCX) [file pbio.3000191.s015.docx]

| **Name** | **Species** | **Sampling Date** | **Address** | **Latitude** | **Longitude** |
| --- | --- | --- | --- | --- | --- |
| G1 | *Prunus mira* | May, 2015 | Bowo, Nyingchi, Tibet, China | 29.90076129 | 95.61345766 |
| G2 | *Prunus mira* | May, 2015 | Bowo, Nyingchi, Tibet, China | 29.90076129 | 95.61345766 |
| GZ | *Prunus mira* | May, 2015 | Bayi, Nyingchi, Tibet, China | 29.700283 | 94.342676 |
| GL2 | *Prunus mira* | August, 2015 | Bayi, Nyingchi, Tibet, China | 29.59609860 | 94.43756066 |
| PXL | *Prunus persica* | Leaf: September, 2015; Root: February, 2016 | Qixia, Nanjing, Jiangsu, China | 32.11544575 | 118.9523901 |
| NJAU1 | *Prunus persica* | April, 2016 | Xuanwu, Nanjing, Jiangsu, China | 32.035682 | 118.845936 |
| NJAU2 | *Prunus persica* | Leaf: April, 2016; Fruit: June, 2016 | Xuanwu, Nanjing, Jiangsu, China | 32.035096 | 118.845313 |
| HY1 | *Prunus persica* | Leaf: April, 2016 and May, 2017; Flower: March, 2017 | Qixia, Nanjing, Jiangsu, China | 32.10859321 | 118.9550963 |
| HY2 | *Prunus persica* | Leaf: April, 2016;  Flower: March, 2017 | Qixia, Nanjing, Jiangsu, China | 32.10855898 | 118.9550291 |
| Maoping | *Prunus persica* | April, 2015 | Ziyuan, Guilin, Guangxi, China | 26.01563 | 110.531881 |
| MHG1 | *Prunus mume* | Leaf: October, 2015; Fruit: May, 2016; Root: July, 2016 | Xuanwu, Nanjing, Jiangsu, China | 32.05080023 | 118.8313390 |
| MHG2 | *Prunus mume* | October, 2015 | Xuanwu, Nanjing, Jiangsu, China | 32.046647 | 118.835417 |
