# Supplementary material for: The architecture of intra-organism mutation rate variation in plants
Source: PLoS Biol. 2019 Apr 9;17(4):e3000191. doi: 10.1371/journal.pbio.3000191 (PMC6456163; doi:10.1371/journal.pbio.3000191)
Supplement: S8 Table — (DOCX) [file pbio.3000191.s016.docx]

| **Individual** | **Valid Sites*** | **Uncallable Sites** | | | | | **Callable Sites** | | | **Fractions of Callable Sites (%)** |
| --- | --- | --- | --- | --- | --- | --- | --- | --- | --- | --- |
|  |  | **Not Called in any Sample** | **Low Depth** (<5)** | **Low Quality (<50)** | **Strand Bias** | **Control samples ungenotyped (≥5)** | **Recovered** | | **Lost** |  |
|  |  |  |  |  |  |  | **Confidence Set** | **Evaluation Set** |  |  |
| G1 | 976 | 268 | 75 | 22 | 27 | 0 | 560 | 22 | 2 | 59.8 |
| G2 | 975 | 270 | 60 | 11 | 20 | 0 | 600 | 14 | 0 | 63.0 |
| GL2 | 976 | 250 | 42 | 21 | 32 | 0 | 617 | 11 | 3 | 64.7 |
| GZ | 978 | 262 | 54 | 18 | 22 | 0 | 612 | 10 | 0 | 63.6 |
| PXL(Leaf) | 991 | 150 | 50 | 21 | 19 | 0 | 729 | 20 | 2 | 75.8 |
| PXL(Root) | 994 | 127 | 67 | 8 | 22 | 0 | 764 | 5 | 1 | 77.5 |
| Maoping | 991 | 170 | 51 | 10 | 11 | 0 | 747 | 2 | 0 | 75.6 |
| NJAU1 | 991 | 109 | 24 | 11 | 9 | 0 | 830 | 8 | 0 | 84.6 |
| NJAU2 | 997 | 210 | 55 | 26 | 19 | 0 | 680 | 7 | 0 | 68.9 |
| HY1 | 995 | 176 | 53 | 26 | 16 | 0 | 721 | 3 | 0 | 72.8 |
| HY2 | 993 | 74 | 18 | 6 | 13 | 0 | 865 | 14 | 3 | 88.8 |
| DHQ1 | 992 | 88 | 23 | 4 | 24 | 0 | 828 | 20 | 5 | 86.0 |
| MHG1(Leaf) | 989 | 180 | 33 | 9 | 28 | 0 | 731 | 8 | 0 | 74.7 |
| MHG1(Root) | 979 | 215 | 74 | 7 | 33 | 1 | 628 | 21 | 0 | 66.3 |
| MHG2 | 985 | 168 | 33 | 7 | 36 | 0 | 732 | 7 | 2 | 75.2 |
| FH1(Leaf) | 992 | 201 | 29 | 28 | 17 | 0 | 680 | 34 | 3 | 72.3 |
| FH1(Runner) | 992 | 205 | 30 | 28 | 17 | 0 | 675 | 34 | 3 | 71.8 |
| WD2(Leaf) | 1000 | 34 | 8 | 1 | 3 | 0 | 931 | 23 | 0 | 95.4 |
| WD2(Root) | 999 | 17 | 4 | 0 | 1 | 1 | 939 | 35 | 2 | 97.7 |
| YAF1(Leaf) | 979 | 115 | 300 | 3 | 46 | 13 | 445 | 57 | 0 | 51.3 |
| YAF1(Root) | 986 | 82 | 407 | 1 | 39 | 8 | 402 | 47 | 0 | 45.5 |
| NIPB | 1000 | 23 | 5.0 | 7 | 11 | 0 | 903 | 6 | 0 | 90.9 |

*Sites overlapped with pre-existing SNV of same allele or indels were considered as invalid; **Only reads with a mapping quality ≥ 20 were counted; ***Synthesized point mutations resided in homopolymer or tandem repeat regions with indels around have a chance to be called as indels
