# Supplementary material for: The architecture of intra-organism mutation rate variation in plants
Source: PLoS Biol. 2019 Apr 9;17(4):e3000191. doi: 10.1371/journal.pbio.3000191 (PMC6456163; doi:10.1371/journal.pbio.3000191)
Supplement: S9 Table — (DOCX) [file pbio.3000191.s017.docx]

| **Individual** | **Valid Sites** | **Sites Filtered** | | | | | | | | **Sites Recovered** | **Fractions of Callable Sites (%)** |
| --- | --- | --- | --- | --- | --- | --- | --- | --- | --- | --- | --- |
|  |  | **Not Called in any Sample** | **Low Depth (<5)** | **Low Quality (<50)** | **Strand Bias** | **Control samples ungenotyped** | **Called only by UG** | **Mimic Reads (≥2)** | **Error Assignment*** |  |  |
|  |  |  |  |  |  |  |  |  |  |  |  |
| Col17 | 1000 | 43 | 33 | 5 | 6 | 50 | 1 | 9 | 189 | 664 | 66.4 |
| Col24 | 1000 | 52 | 29 | 4 | 13 | 37 | 2 | 11 | 190 | 662 | 66.2 |
| DG1(Leaf) | 990 | 262 | 25 | 28 | 31 | 0 | 5 | 2 | 61 | 576 | 58.2 |
| DG1(Root) | 992 | 287 | 252 | 11 | 18 | 17 | 2 | 0 | 85 | 320 | 32.3 |
| PA1 | 990 | 200 | 36 | 29 | 22 | 2 | 3 | 1 | 65 | 632 | 63.8 |
| KA1 | 991 | 258 | 41 | 27 | 25 | 2 | 3 | 2 | 35 | 598 | 60.3 |

*Samples with no more than two synthetic reads would be wrong-assigned as control samples which lead to the rejection of this site.
